# Spatial dynamics and control of a crop pathogen with mixed-mode transmission

**DOI:** 10.1371/journal.pcbi.1005654

**Published:** 2017-07-26

**Authors:** Christopher Finn McQuaid, Frank van den Bosch, Anna Szyniszewska, Titus Alicai, Anthony Pariyo, Patrick Chiza Chikoti, Christopher Aidan Gilligan

**Affiliations:** 1 Computational and Systems Biology, Rothamsted Research, West Common, Harpenden, United Kingdom; 2 Root Crops Research Programme, National Crops Resources Research Institute, Namulonge, Kampala, Uganda; 3 Zambia Agriculture Research Institute, Plant Protection and Quarantine Division, Mt. Makulu Research Station, Chilanga, Zambia; 4 Department of Plant Sciences, University of Cambridge, Cambridge, United Kingdom; Ecole Normale Supérieure, FRANCE

## Abstract

Trade or sharing that moves infectious planting material between farms can, for vertically-transmitted plant diseases, act as a significant force for dispersal of pathogens, particularly where the extent of material movement may be greater than that of infected vectors or inoculum. The network over which trade occurs will then effect dispersal, and is important to consider when attempting to control the disease. We consider the difference that planting material exchange can make to successful control of cassava brown streak disease, an important viral disease affecting one of Africa's staple crops. We use a mathematical model of smallholders’ fields to determine the effect of informal trade on both the spread of the pathogen and its control using clean-seed systems, determining aspects that could limit the damage caused by the disease. In particular, we identify the potentially detrimental effects of markets, and the benefits of a community-based approach to disease control.

## Author summary

Cassava brown streak disease affects yields of cassava, an important subsistence crop in Uganda. Relatively little is known about the disease and how it spreads across a landscape, especially regarding the importance of trade in moving infected planting material. Despite this, a number of different strategies are being implemented in order to control the disease. However, the interaction between these strategies has not been considered and failure to do so could have detrimental consequences; for example, restricting trade may reduce the spread of disease through planting material, but may simultaneously reduce the spread of improved, disease-free planting material. Here we use mathematical models to assess the potential importance of trade in spreading cassava brown streak disease. We consider what happens to infection levels when trade is restricted, and how trade restrictions can be combined with the use of clean seed systems, which distribute disease-free planting material to growers. The results and recommendations from our work are of practical use to inform strategies for the control of cassava brown streak disease, and more generally in assessing the effectiveness of clean seed strategies.

## Introduction

The scales over which pathogens are transmitted are of vital importance in disease control [1], particularly when multiple modes of transmission are possible. The simultaneous use of different control strategies targeting different transmission modes could have an antagonistic effect, whereby one control undermines another. For example, a restriction on movement of planting material to reduce the spread of infection may impact the distribution of disease-free material, affecting efforts to reduce dissemination of pathogen by a vector. Control strategies, therefore, need to be carefully matched to all modes of transmission in order to optimise the effectiveness of disease reduction.

A case in point is cassava brown streak disease (CBSD), one of the most economically important viral diseases affecting cassava in sub-Saharan Africa. Viral infection causes necrosis of the edible tuberous roots, leading to yield and quality loss [2]. The CBSD viral pathogens are *Cassava brown streak virus* (CBSV) and *Ugandan cassava brown streak virus*, both in the genus *Ipomovirus* and family *Potyviridae*, and often together referred to as cassava brown streak viruses (CBSVs, see [3, 4]). The pathogens are transmitted through infected planting material as well as non-persistently by a whitefly vector, *Bemisia tabaci* [5–8], and possibly through other hosts and vectors as well [6, 9, 10].

Informal trade or sharing of cassava planting material, which may carry the pathogen, is important to subsistence growers as a cheap means of obtaining what is otherwise perceived to be superior material. Informal trade or sourcing of stem cuttings (here considered to refer to any form of movement of material between growers) is difficult to regulate, but the movement of infected material through this trade is thought to be important in long-distance pathogen dispersal [11].

One strategy to reduce infection within fields that also reduces the movement of infected planting material between fields as a secondary by-product, involves the deployment of certified virus-free cuttings for planting, referred to as a “clean-seed” system [6, 9, 12]. Typically, clean-seed systems target new plantings or replanting of fields by growers. Early-generation planting material (‘seed’) is developed by breeders and sold, after bulking up in basic seed fields, to cassava seed entrepreneurs, who multiply material for sale. Interest in this approach is growing, and organisations in Uganda and Tanzania are in the process of establishing clean-seed systems [11, 13].

Clean-seed systems for CBSD are currently at an empirical stage of planning, and data have yet to emerge on their effectiveness. Accordingly, we use modelling to integrate current knowledge about the disease and its control in order to compare the effectiveness and risks of failure of different strategies for the introduction of clean-seed systems. Results of this study can then be used to guide the selection of strategies likely to be successful and to avoid those that carry a significant risk of failure.

In order to explore scenarios for the introduction of a clean-seed system effectively, it is first necessary to understand the dynamics of the disease. Cassava is grown on a spatial network of fields, where the pathogen is spread through the movement of whitefly and planting material. We consider two hypotheses: the first is that the movement of planting material through trade is more important than dispersal of the vector in affecting long-range dispersal of the pathogen (H1). Our second hypothesis is that the nature of the network over which planting material is moved affects the rate of dispersal: in particular, fewer connections and greater consistency of connections over time is expected to slow dispersal (H2).

We address the optimisation of the introduction of a clean-seed system within a trade network to maximise disease reduction. Our use of ‘trade network’ in this context refers to a simple summary of where growers access cassava cuttings from, determined by a stochastic dispersal kernel. We do not analyse here the social drivers that determine grower behaviour. We hypothesise that restrictions or reductions in trade slow the dispersal of the pathogen more than the distribution of clean planting material, and so reduce the rate of pathogen spread overall (H3). We also hypothesise that a community-based approach to clean-seed use, where an entire group receives material rather than a number of spatially-dispersed individual growers, protects fields from reinfection from neighbouring fields and hence decreases disease incidence to a greater extent than a random approach to the distribution of clean planting material throughout the landscape (H4).

We model the spread of a single strain of non-evolving CBSV both within and between growers’ fields, where the host distribution is characterised by that of Nakasongola district in central Uganda. The district suffers from high levels of CBSD infection, where a clean-seed system might have a positive impact. We firstly consider pathogen dispersal from one initially infected field when the district was disease-free, and secondly when the disease is endemic within the district and a clean-seed system is introduced.

## Results

We consider two cases:

- The effects of trade patterns (whereby we consider the numbers of trade partners for acquiring stem cuttings and the consistency of these partnerships from season to season) on the rate of spread of the pathogen and hence the disease into a previously disease-free area (H1 and H2);
- How to target cuttings amongst a population of growers when introducing a clean-seed system to an area where disease is endemic (H3 and H4).

Additionally, we make the following definitions:

- *Loyal growers* are those who obtain planting material from the same sources (growers) over successive seasons.
- *Disloyal growers* are those who obtain planting material from different sources every season.
- *Fixed distribution* refers to the distribution of clean planting material to the same growers over successive seasons.
- *Varied distribution* refers to the distribution of clean planting material to different growers every season.

### The effect of planting material trade and whitefly dispersal

**Fig 1:**
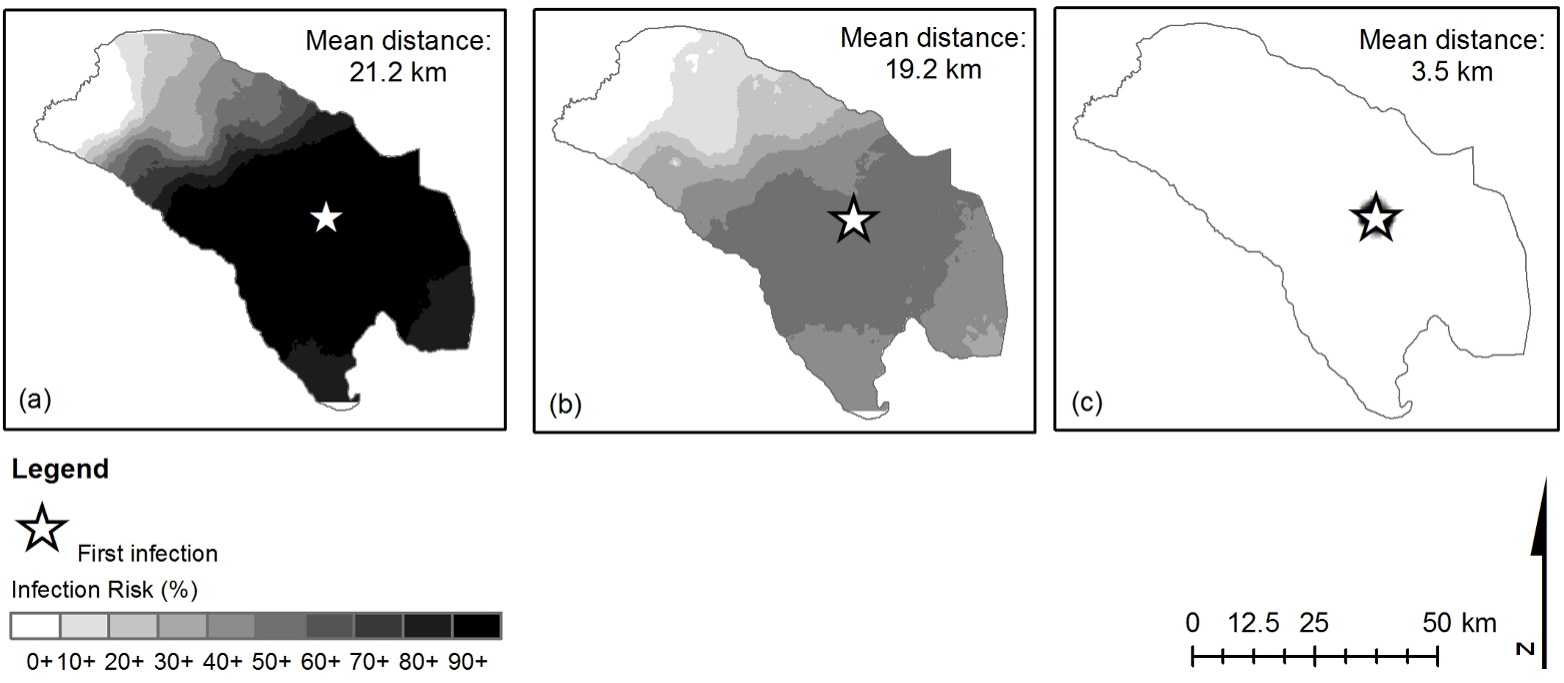
Disease dispersal across the district. Model predictions of the dispersal of CBSVs in Nakasongola district after 30 seasons from an initially infected source field located at the white star. Dispersal of the pathogen between fields occurs through (a) both the trade of infectious planting material and the between-field dispersal of infectious whitefly, (b) trade only, with within-field dispersal of whitefly or (c) between-field dispersal of whitefly only, where the scale shows the risk of becoming infected during an epidemic for a field at any given point. The results show the average infection from 100 realisations of the model, where the mean distance refers to the average distance over all realisations from the source of infection to all infected fields. Parameter values are summarised in Table 1.

**Fig 2:**
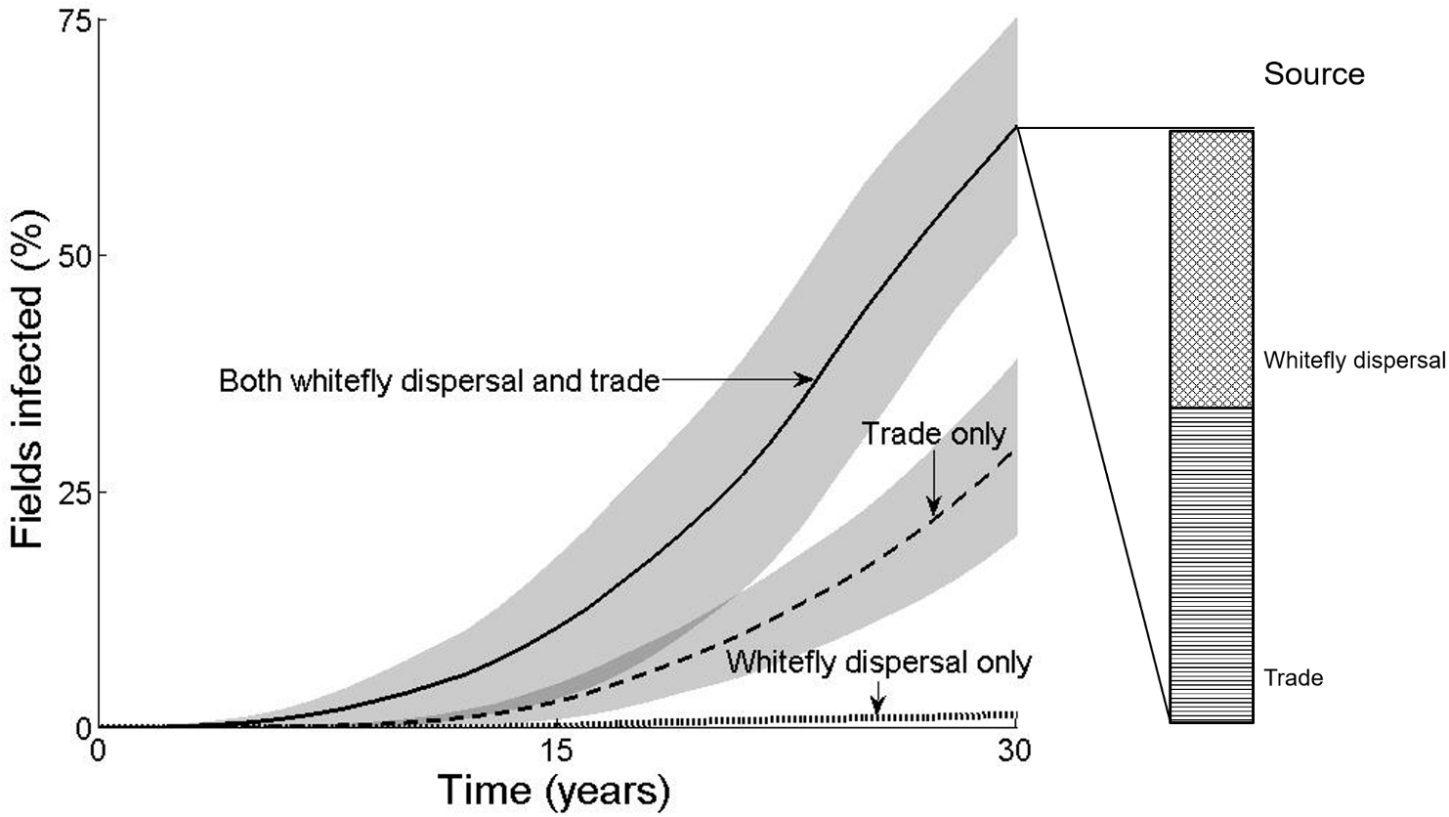
Number of fields infected. Model predicitons for the percentage of fields in Nakasongola district that are infected after 30 seasons. Dispersal of the pathogen between fields occurs through both the trade of infectious planting material and the between-field dispersal of infectious whitefly (solid line), trade only (dashed line, with within-field dispersal of whitefly) or between-field dispersal of whitefly only (dotted line). Light grey areas represent one standard deviation around the mean from 100 realisations of the model, while dark grey areas (cf. 15 seasons) represent the overlap of standard deviations. To the right of the plot a bar represents the source of infection for fields in the case of both trade and between-field dispersal; i.e. the percentage of infected fields after 30 seasons that were initially infected for the first time by the trade of infectious material compared with those that were infected by the between-field dispersal of an infectious whitefly from outside the field.

We begin in Fig 1 by removing either trade or between-field whitefly dispersal as a means of pathogen dispersal, and see that under current estimates trade (Fig 1B) is important in effecting long-range dispersal of the pathogen. By contrast, between-field whitefly dispersal alone (Fig 1C) does not result in epidemics on a large scale. However, without between-field whitefly dispersal, nearly half (47%) of all epidemics fade out, as the pathogen fails to take hold in the district. This is a result of stochasticity in the network of trade interactions, and reflects the disappearance of the pathogen from the system when incidence is low and all infected cuttings are removed but not replanted at the end of a season. Although the results displayed are conditioned on a lack of fade out, to avoid skewing the data, it is important to bear these failed epidemics in mind. Our result show that, while trade alone results in many more infected fields than between-field whitefly dispersal alone (Fig 1), when both means of pathogen dispersal are taken together, local transmission between fields by whitefly movement accounts for nearly 50% of newly infected fields (Fig 2). This is likely due to the fact that whitefly movement is faster on a small spatial scale; in our model whitefly disperse continuously between fields while trade is seasonal (see, for example, S2 Appendix, where new infections through trade events occur over large distances followed by multiple new infections over small distances through whitefly movement). See S1 Video–S8 Video for video files demonstrating the above.

**Fig 3:**
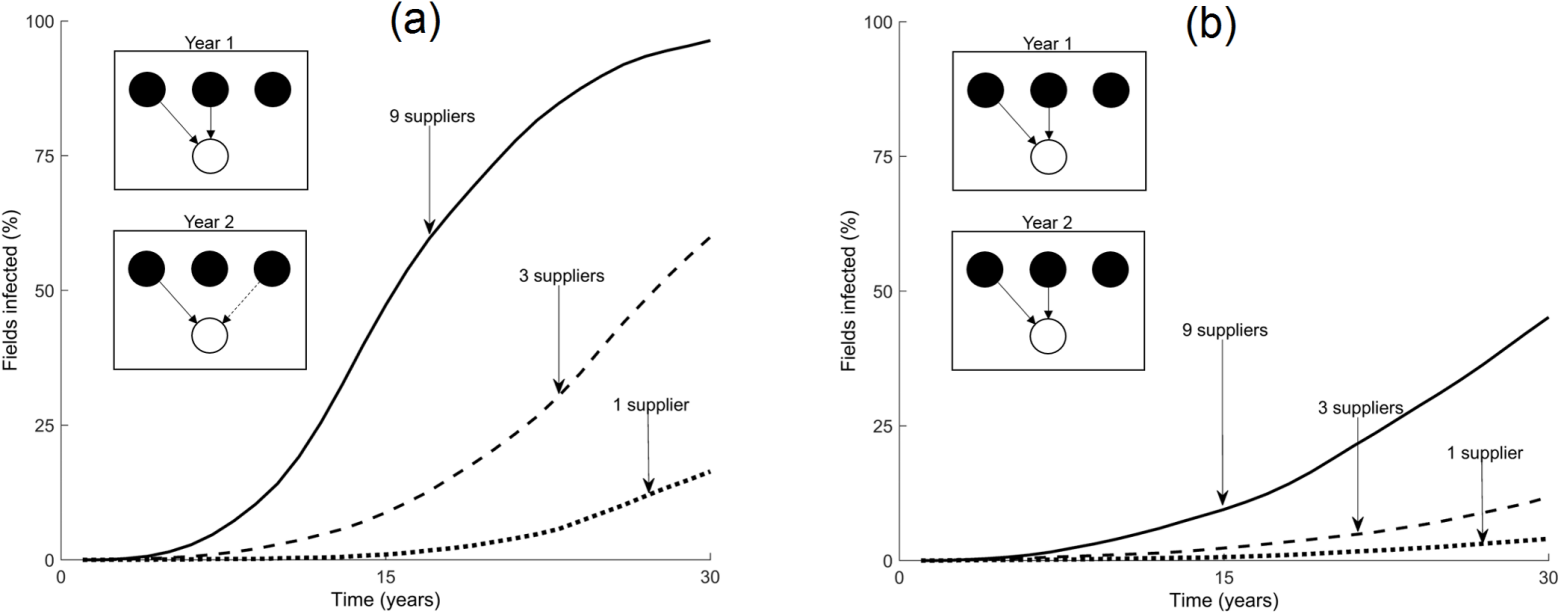
The effect of grower loyalty. Model predictions for the percentage of fields in Nakasongola district that are infected after 30 seasons, when growers are (a) loyal to suppliers, obtaining material from the same sources every season, or (b) disloyal to suppliers, obtaining material from different sources every season. Legends display the maximum number of suppliers from whom a grower may obtain cuttings, where the number of suppliers is drawn from a uniform distribution. Results are the mean of 100 realisations of the model each. Examples of loyal and disloyal growers are shown in the insets. The black circles represent suppliers of planting material for growers, represented by white circles. Arrows depict trade events, following the movement of planting material to a grower. In (a) the grower is loyal, while in (b) the grower is disloyal; after one season, the grower changes one of the sources of her material (dashed arrow), representing her lack of loyalty to trading partners.

We now change the loyalty of growers to suppliers of planting material, as well as varying the maximum number of suppliers for a grower (Fig 3). The rate of pathogen spread is reduced when growers continue to trade over successive seasons with the same partners, and when the number of trading partners is reduced. Hence, as connectivity in the trade network increases, and the average path length (average shortest number of steps between any two growers, in terms of trading events) decreases, so does the rate of spread of the pathogen. This further influences the risk for an individual grower of obtaining infected material, which follows a similar pattern to that of the population (see S2 Appendix for further details).

### The introduction of a cassava clean-seed system when CBSD is endemic

We consider the introduction of a clean-seed system as well as restrictions on trade to Nakasongola district when CBSD is both endemic and near-ubiquitous (a 2013 survey showed that 70% of fields had CBSD with 100% incidence in a field). While trade between growers could promote the spread of clean material, this has to be balanced against the potential for trade to favour the spread of the pathogen. Here we compare yield, as opposed to the number of fields infected or the average infection, as this is a more relatable measure of success for a control strategy. Yield is measured as the percentage of uninfected or latently infected plants harvested, together with a percentage (here 30%) of each infectious plant that is undamaged by necrosis (and therefore usable). Hence, yield decreases linearly with the increase of infection in the district (see, for example, [14, 15]).

**Fig 4:**
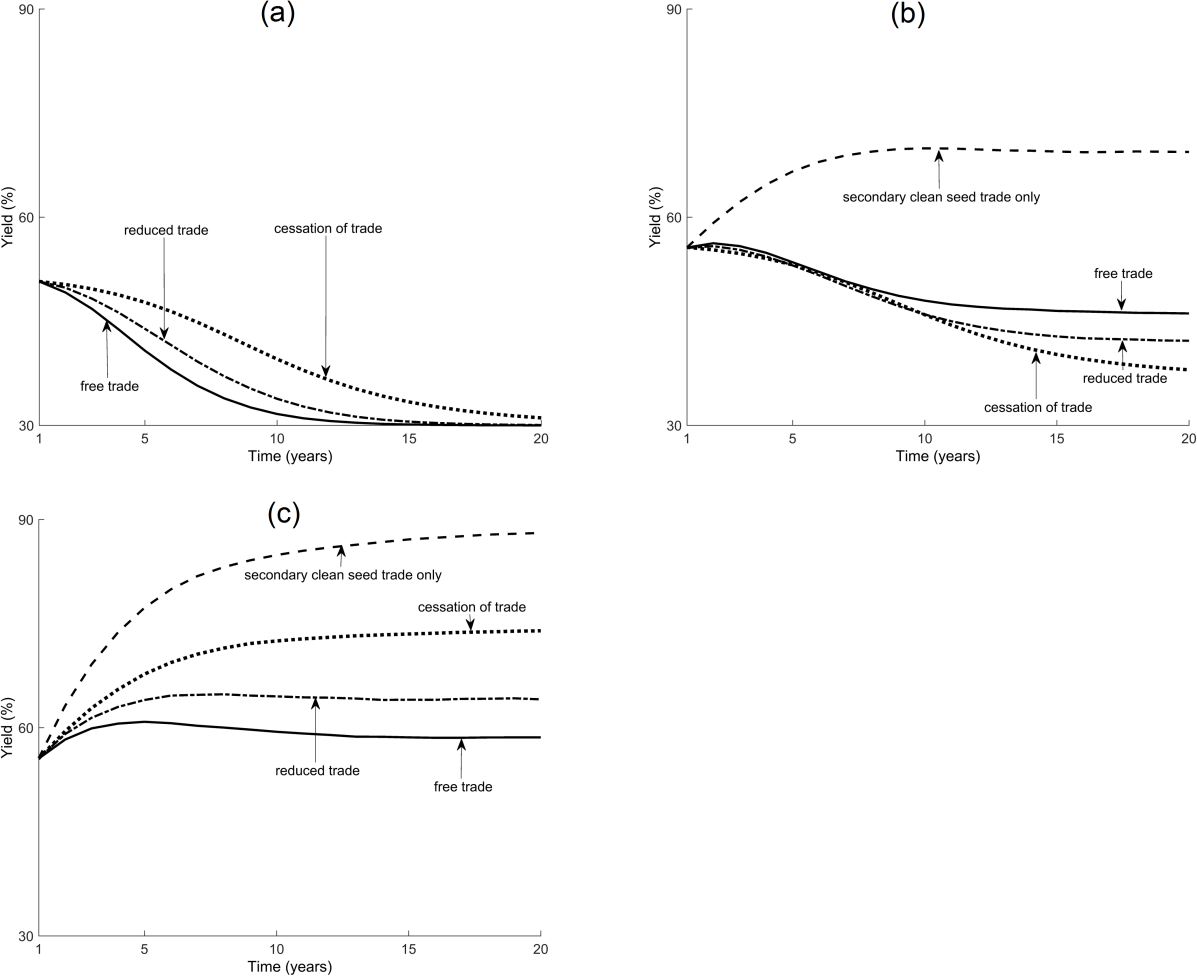
The effect of trade restrictions. Model predictions for average yield of cassava production in Nakasongola district after 20 seasons, where 70% of fields are infected with incidence of 100%. Clean planting material from a clean-seed system is used by (a) none of the growers, (b) 10% of the growers, distributed to the same growers over successive seasons or (c) 10% of the growers, distributed to different growers every season. Trade in planting material is allowed to continue as usual (solid lines), is reduced by 50% (dot-dashed lines), completely ceases (dotted lines) or only users of certified clean material are licensed to distribute material (dashed lines). Each line is the mean of 100 realisations of the model.

In Fig 4 we consider the introduction of a clean-seed system, with planting material either distributed to the same growers each season or to different growers. We also introduce a set of trade restrictions: trade in planting material is allowed to continue as usual; trade is reduced by 50%; trade completely ceases (with between-field transmission restricted to whitefly dispersal); trade is restricted so that only users of certified clean material in a particular season are licensed to distribute material at the end of that season.

Our results show that clean-seed systems can work and increase yield; every case where the system is implemented results in higher yields than where it is not (cf Fig 4B and 4C with Fig 4A). The effect of restrictions on trade, however, depends on how the clean planting material is distributed. If the virus-free material is distributed to the same growers every season, restricting trade (i.e. reducing the frequency of trading events and numbers of trading partners) decreases the yield throughout the district (Fig 4B). This is because only a few growers benefit from the clean material while any remaining uninfected fields are susceptible to infection from dispersal of infectious whitefly. In comparison, if trade were to continue as usual, neighbours of users of the clean-seed system would benefit from the system indirectly by trading for relatively clean material from those users. When virus-free material is distributed to different growers every season, restricting trade increases the yield throughout the district (Fig 4C), as the material still reaches a large number of growers, while the rate of pathogen dispersal is also decreased through the trade restrictions (Fig 3). Finally, restricting trade such that only the beneficiaries of clean planting material may sell the planting material that they obtain at the end of a season always increases the yield throughout the district (Fig 4B and 4C). Effectively, this strategy promotes the trade of relatively clean material from seed system users, but discourages trade in potentially diseased material from growers that do not use the system. The result holds even if the seed system material contains some infection (see S2 Appendix).

**Fig 5:**
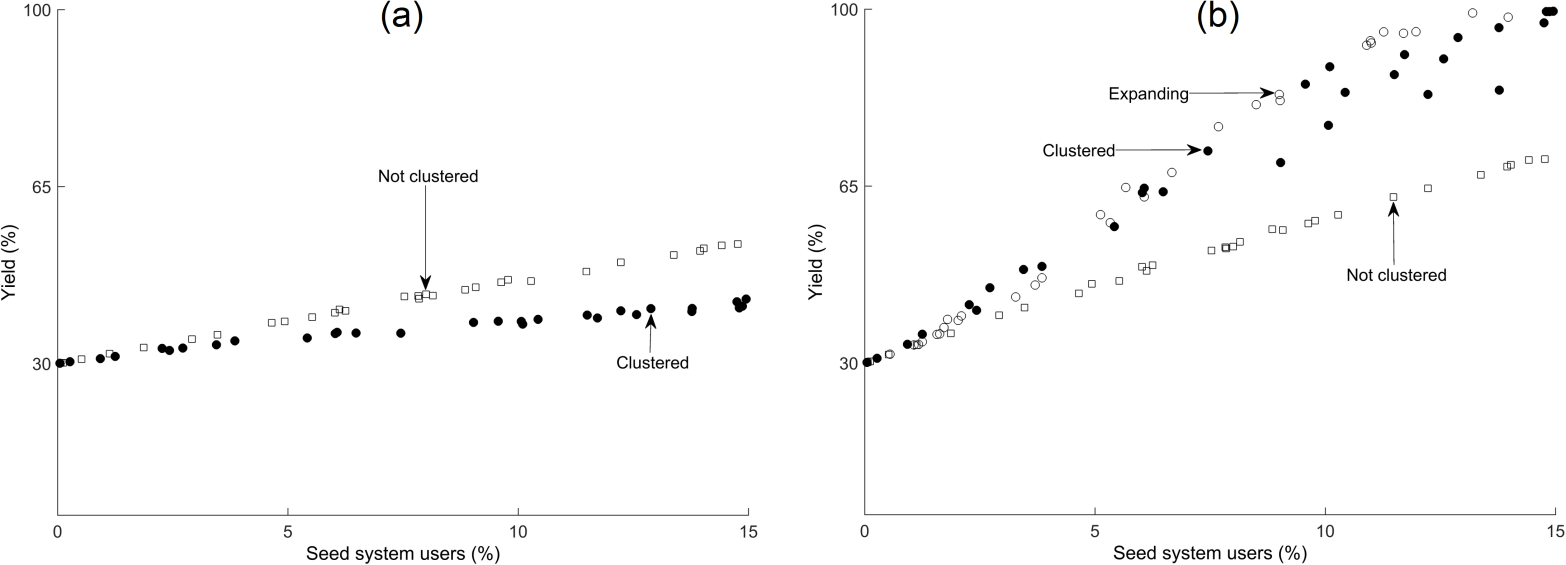
The effect of clean seed dispersal mechanism. Model predictions for average yield of cassava production in Nakasongola district after 20 seasons, where 70% of fields are infected with incidence of 100%, for varying proportions of growers using certified clean planting material. Clean planting material is distributed in (a) a fixed manner, to the same growers over successive seasons, or (b) a variable manner, to different growers each season. Material is distributed either to a cluster of growers (circles) or at random (open squares). In (b) the material is distributed either to a different cluster of growers each season (filled circles) or to a different group of growers each season expanding outwards from an initial cluster (open circles). Results are from 30 realisations of the model for disloyal trade across the district, but are qualitatively similar for loyal trade.

We now consider the manner in which clean planting material is distributed to growers; whether individuals or entire communities are targeted and whether these are supplied for only one season or resupplied over a number of seasons. Our results show that regional yield is enhanced, in ascending order, by:

- supplying clean planting material to the same clusters of growers in successive seasons (Fig 5A, similar to a targeted community approach)
- supplying clean planting material to the same group of randomly distributed growers in successive seasons (Fig 5A, similar to the supply of material by extension workers, who often work with a fixed group of amenable growers)
- supplying clean planting material to different groups of randomly distributed growers every season Fig 5B, similar to the sale of material through markets, or the approach taken by the Great Lakes Cassava Initiative project which supplied a small amount of material to a widespread number of growers, see [16])
- supplying clean planting material to different clusters of growers every season ([Fig 5B, similar to the use of a travelling salesman, or the apporach of the Mennonite Economic Development Associates who look to subsidise different growers each season to sell clean material to their neighbours, see [17])
- supplying clean planting material to a different group of growers every season expanding outwards from an initial cluster of growers, when the number of users is high (Fig 5B, similar to the expanding community approach of the Community Phytosanitation project, see [10])

Note that when material is distributed to the same group of clustered growers every season, our model shows that decreasing the number of individuals in each cluster increases the yield, until each cluster consists of a single grower only and the material has essentially been randomly dispersed. The reverse is true if the material is distributed to different growers each season. Yield increases faster to a higher equilibrium level when clean planting material is distributed to different growers every season, extending the reach of the material rather than ensuring the success of only a few growers (see Fig 5).

Finally, we note two important asides; firstly, our results are qualitatively consistent (S2 Appendix) if the material distributed is tolerant or resistant to disease (here we presume this affects the useable proportion of yield from an infectious plant or the rate of infection, *β*_*p*_, respectively). Secondly, our results are consistent for different agronomic systems, including different sizes and densities of fields, different whitefly populations and annual versus continuous sowing and harvest (S2 Appendix).

## Discussion

### H1: Planting material trade and whitefly dispersal

Given the assumptions about the relative magnitudes of trade and vector dispersal, we have shown that trade is the key long-distance pathogen dispersal mechanism, although it also contributes to spreading the pathogen within a community. This is consistent with suggestions that trade has been responsible for introductions of the pathogen to previously uninfected areas [7, 13]. However, trade may also help to eradicate the disease during the early stages of an epidemic if growers actively select healthy planting material from neighbours, but only if whitefly numbers are very low. Our results show that whitefly transmission is of key importance in local amplification, dispersing the pathogen from new sources of infection to fields immediately surrounding them (see, for example, S2 Appendix). The potential for local spread of infection is a particular problem given significant recent increases in whitefly populations [7]. While whitefly dispersal alone spreads the pathogen slowly, it does ensure that the disease remains endemic. This implies that the success of a control strategy in controlling an epidemic depends upon how the strategy interacts with both trade as well as the vector.

### H2: The nature of the trade network

Our results indicate that the nature of the network of trade interactions is important to disease incidence, as expected for any such disease (see, for example, [18, 19]). Disease incidence is lower when growers consistently use the same suppliers for cuttings, as well as when growers have fewer suppliers (see also [20, 21]). If trade is restricted to a few, constant trading partners, it is possible that disconnected sub-networks of growers with highly variable levels of infection could be created, into or out of which the pathogen disperses slowly through between-field whitefly dispersal (see, for example, S2 Appendix). Such restriction in trade, combined with low whitefly numbers and high levels of replanting from the same field, may well be slowing the arrival of CBSVs into areas such as Eastern Zambia. In fact, in areas where growers are aware of the disease, were they to decrease the number of suppliers from whom they obtained material they would decrease their likelihood of acquiring infectious material. We note that this is seldom likely to be the case, however, as growers are often unaware of either the disease or its transmission mechanisms [22, 23].

### H3: The introduction of trade restrictions

Separating out the two forms of between-field disease dispersal allows us to evaluate the effect of control on each dispersal type separately, in an approach that could be readily adapted to diseases such as bluetongue disease (see, for example, [24]). Reducing informal trade in conjunction with the introduction of a clean-seed system is only effective when clean planting material is distributed to different growers every season, in which case we would suggest encouraging growers to reduce exchange of planting material by trade (see, for example, [11]). Practical difficulties with encouraging such a change in practice would be challenging, but a reduction in trade only, rather than a complete cessation, would still be beneficial. However, while a reduction in trade may reduce disease across the district as a whole, it may also lead to local shortages of clean planting material, which would be less likely if trade were allowed to continue as normal. Promoting users of clean planting material to trade their material increases yield the fastest and to a higher level through the secondary dispersal of clean planting material.

### H4: The introduction of a clean-seed system

We considered various types of community-based approach for the introduction of a clean-seed programme. Distributing uninfected planting material to different growers each season increases the probability of success in reducing yield loss (see, for example, [10, 11, 25]). However, ancillary challenges in encouraging uptake by growers as well as managing logistical constraints associated with bulkiness and perishability of cassava planting material would need to be addressed (see [13, 26]).

The following principles emerged in considering how to improve the effectiveness of clean seed systems;

- protecting fields from reinfection by reducing infection in neighbouring fields
- protecting fields from reinfection by resupplying their owners with clean material
- reaching more growers through widespread distribution of users, who trade with their (non-user) neighbours
- reaching more growers through a change in users each season

Reaching more growers is vital to preventing a bottleneck in distribution, hence the success of approaches which supply different growers each season. This can be combined with protection through community action, where an entire community is supplied with material, to slow reinfection. The coordination involved in the expanding approach further improves reach by ensuring that every grower is supplied at some point, although it does this at the expense of a certain degree of protection from reinfection through community action. The effect is particularly visible when the number of users is small; growers who receive material later in the program, while they may have neighbours who previously used the clean-seed system and hence have relatively low incidence, are likely to be widely dispersed with few immediate neighbours still using the system. See S1 Video and S4 Video–S8 Video for video files demonstrating the above. We note that these general conclusions could be applicable to other vector- and trade-borne infections, highlighting the need for a balance between community action and the prevention of bottlenecks in distribution.

In terms of distributing clean planting material to growers, we relate our proposed strategies to those that are currently implemented by a variety of organisations. A focused community-based project that does not expand with time, is the least effective method of distribution, although it might be the simplest in terms of costs. Distribution of material to the same group of dispersed growers in successive seasons describes the traditional approach taken by extension workers, working with particular amenable growers. This traditional approach has the potential to reach a greater number of recipients indirectly, although it does decrease the protection that a community of growers using clean planting material confers. In comparison, distributing uninfected planting material to a number of growers that changes across a widespread area (such as the approach taken by the Great Lakes Cassava Initiative project, see [16]) or through sale at a market, reaches a still larger number of growers. However, integrating the changing cohort of growers within a community-based approach significantly increases the effectiveness of a clean-seed programme by extending the reach while also reducing transmission of infection via planting material. The approach is similar to the concept of using a travelling salesman who sells clean planting material to a cluster of growers in one season, moving to a new group in the next. When a high proportion of growers use clean material, an expanding community approach to selling material to growers is optimal: each season uninfected planting material is sold to a different group of growers, expanding outward from an initial group of users.

Of course, these results do not take into account the behaviour of individual growers, or the costs associated with different strategies. Recent work has demonstrated that grower perception of risk and reward can significantly affect the success of control for disease in agriculture [27], an aspect that has long been of interest in human disease studies [28]. At the same time, the cost of applying a particular control option must be weighed against the benefit that it would bring, such that epidemiological considerations alone are not sufficient cause to utilise a given strategy (see [29–31]).

Additional uncertainty analyses (S2 Appendix) show that our results are qualitatively robust to model assumptions (such as the nature of the migration and dispersal of whitefly) and also hold for different agronomic situations, including those with high or low whitefly density (and by extension disease pressure), discrete planting and harvesting (commercial growers), discrete planting and continuous harvesting (casual growers) or continuous planting and harvesting (subsistence growers) cycles, different sizes of fields (and by extension commercial, casual or subsistence growers in rural or urban environments) and different densities of fields (areas more or less dependent on cassava). These findings are therefore applicable to other locations across Uganda and the region, although the access for certified seed producers located within the district to basic seed from low disease-pressure areas is important in ensuring that the clean-seed remains disease-free.

Our results also show that a community-based approach to manage the exchange of material between different growers over successive seasons would increase the probability of success for the introduction of partially-resistant or tolerant varieties (S2 Appendix). Care would need to be taken to monitor the effects of partial resistance on pathogen build-up as well as the risks of unintended consequences such as distributing varieties with increased susceptibility to other cassava diseases such as cassava mosaic disease [7, 12, 25, 32]. The probability of success in limiting disease spread and crop loss would be enhanced by augmenting a clean-seed scheme with roguing of infected plants and isolation of uninfected fields [13].

We conclude that trade, informal or otherwise, leads to long-range pathogen dispersal, and whitefly to local amplification. Restrictions on trade can therefore reduce pathogen dispersal. Unrestricted trade via markets favours the spread of the pathogen in a new area by promoting exchange between many, changing partners over time. Combining a general reduction in trade with the encouragement of growers using certified clean planting material to trade is always beneficial. In an example of this, Uganda is currently piloting the establishment of a functional seed system for cassava with the introduction of an “agricultural police service” to monitor the movement of planting material. Were this unit to be effective in reducing trade in infected material, it would aid in significantly increasing yields.

A project supported by the Bill and Melinda Gates Foundation in Tanzania, entitled Community Phytosanitation, is currently in the early stages of using a whole-community approach to reduce disease through clean planting material and roguing (which we do not include here, but which could further decrease infection in fields), with continuous expansion outwards from these communities to neighbouring groups [10]. Similarly, the Canadian Mennonite Economic Development Associates [17] are attempting to set up clean-seed systems in Tanzania with a focus on cassava seed entrepreneurs who sell material locally, again aiming to target different entrepreneurs for subsidy each season, which will lead to access to clean planting material for different groups of growers. Our results suggest that both approaches are likely to be effective (in particular, more so than previous strategies) for disease reduction when distributing clean material, but only as long as the strategies focus on continued expansion with infrequent replenishment over continuously resupplying past users.

## Methods

The infection level in each field is calculated using a mean-field ordinary differential equation model. The rate of spread of the pathogen between fields is calculated from a dispersal kernel for whitefly transmission between fields within season, and from the stochastic trade of planting material at the start of a season.

### Population dynamics

Previously, [33] and [34] constructed models for cassava mosaic disease which considered susceptible and infected plants, as well as infectious vectors. We build on these basic models, adapting them to CBSD as well as making fields spatially explicit and including the movement of vectors and material that this entails. For each field *i* we consider susceptible uninfected (*S*_*i*_), latently infected (*L*_*i*_) and infectious and symptomatic (*I*_*i*_) plants and infectious vectors (*V*_*i*_, with total constant whitefly vector population *W*). For fields *i* = 1 … *N*,

$$\begin{array}{l} \mathrm{Susceptible}\;\mathrm{plants}:\;\frac{dS_{i}}{dt}=\mu \left(1-q_{i}\sum_{j=1}^{N}\tau_{ij}\frac{C_{j}}{P_{j}}\right)-hS_{i}-\beta_{p}S_{i}V_{i},\\ \mathrm{Latently}\;\mathrm{infected}\;\mathrm{plants}:\;\frac{dL_{i}}{dt}=\mu q_{i}\sum_{j=1}^{N}\tau_{ij}\frac{C_{j}}{P_{j}}+\beta_{p}S_{i}V_{i}-\left(h+\gamma_{p}\right)L_{i},\\ \mathrm{Infectious}\;\mathrm{plants}:\;\frac{dI_{i}}{dt}=\gamma_{p}L_{i}-\left(h+g\right)I_{i},\\ \mathrm{Infectious}\;\mathrm{vectors}:\;\frac{dV_{i}}{dt}=\beta_{v}I_{i}\left(W-V_{i}\right)-\left(\lambda +\omega \right)V_{i}+m\left(\sum_{j=1,j\neq i}^{N}\delta_{ij}V_{j}-V_{i}\right). \end{array}$$ (1)

During each discrete season, plants are harvested or rogued and cuttings potentially replanted, while infection occurs through infected cuttings or infectious whitefly. Whitefly are infected upon contact with infectious plants and lose infectivity, die or migrate. We use a density-dependent approach as the contact rate between whitefly and cassava increases with the density of both; the whitefly is more likely to encounter cassava rather than a different plant, while the plant is more likely to encounter a whitefly. This approach also allows for different whitefly population sizes in different fields, which may affect disease dispersal, particularly at the infection front.

Once planted at the beginning of a season, plants are harvested at rate *h*. Additional plants may be replanted at rate *μ*, with material obtained from the grower’s own field, through trade with the owner of another field (*q*_*i*_ = 1), or from a clean-seed system (*q*_*i*_ = 0). When cuttings are not obtained from the grower’s own field, the proportion of cuttings in field *i* obtained from every field *j* is given by *τ*_*ij*_, so that $\sum_{j=1}^{N}\tau_{ij}=1$. In the absence of a comprehensive set of empirical data, we assume that trade networks can be generated through a dispersal kernel describing the probability of planting material movement between fields. For a grower at field *i* trading with neighbours, each neighbouring field *j* is ranked according to $\rho_{ij}=re^{\frac{-d_{ij}}{d_{max}}}$ for *d*_*max*_ the maximum distance between any two fields and random variable *r* ∈ [0,1] taken for simplicity from a uniform distribution, where grower *i* trades with a random number of the highest ranked neighbours from a uniform distribution up to a given maximum. We note that the dispersal kernel implicit in the ranking does not qualitatively affect our results. Dispersal based on both an exploratory data set and a fat-tailed dispersal kernel, resulting in more frequent long-distance transactions, yield similar results. This is likely due to the fact that all assumed trade acts over a significantly larger scale than whitefly dispersal, in part due to the field density. See S3 Appendix for different trade dispersal assumptions. The level of infection in the material obtained from field *j* is determined by *P*_*j*_ and *C*_*j*_, the total number of uninfected and infected plants respectively harvested from field *j* in the previous season. Latently infected harvested plants may also have sufficiently low viral load as to undergo reversion at rate *η*, although no data yet exist for this parameter and we assume it to be zero. In this way, the proportion of infected cuttings replanted depends on whether or not the grower is using a clean-seed system, and the incidence of infection in material that the grower has collected both from her own and her neighbour’s fields. Plants may additionally be infected through contact with infectious vectors, which infect uninfected plants at density-dependent rate *β*_*p*_. Once infected, latently infected plants progress to a fully infectious state at rate *γ*_*p*_, when they may be removed by roguing at rate *g*.

In terms of the vector, non-infectious vectors (*W*−*V*_*i*_) become infectious at density-dependent rate *β*_*v*_ through contact with infectious plants. The vector loses infectivity at rate *λ*, and dies at rate *ω*. Vector dispersal between fields occurs at rate *m*, where we assume emigration and immigration rates are the same. We assume this is constant for emigration, while for immigration into field *i* we sum dispersal of infectious vectors from each field *j* to field *i*. This is given for distance *d*_*ij*_ between the pair of fields by the probability density dispersal function $\delta_{ij}=\frac{A\alpha^{2}}{2\pi }e^{-\alpha d_{ij}}$, for attractive area *A* which measures not only the actual size of the field but also the preference of whitefly for a cassava field over uncultivated land) and mean dispersal distance *α*^−1^. For simplicity, we assume a constant vector population, and do not include seasonal dynamics here.

### The system

Parameter values are summarised in Table 1. The model describes a sequence of dynamics within successive growing seasons; any remaining plants not harvested during the season are harvested at the end of each season and cuttings replanted the following season (where initial conditions are based on the incidence of the previous season). For simplicity, we assume that there is one growing season of 300 days per year, ignoring the initial two months of the season when there is no foliage and whitefly cannot transmit the pathogen, although we note that in reality there may be some transmission between 1 and 2 months. Of course, in reality many different cropping systems are possible across the continent, from subsistence to commercial farming, and from rainy season(s) determining when cassava is planted to continuous planting and harvest. Growing seasons may be staggered in some areas, and fields may be ratooned at different stages. During the initial two months we assume that all whitefly lose infectiousness, due to the brevity of their period of infectiousness [7, 11], and plants progress to a fully infectious state before the disease dynamics become relevant. Hence, all latently infected or infectious cuttings that have been planted are assumed to be fully infectious at the start of each season, and the number of initially infectious whitefly is zero.

**Table 1:** Model parameters and default values.

Parameter	Description	Value^a^	Source
*h*	harvesting rate	0.003 day^-1^	[33]
*μ*	replanting rate	0 plants·day^-1^	See also [34]
*g*	roguing rate	0 day^-1^	-
*β*_*p*_	infection rate of plant	0.007 vector^-1^·day^-1^	[8]
*γ*_*p*_	disease progression rate in plant	0.035 day^-1^	[8, 35]
*η*	reversion ratio	0	-
*β*_*v*_	virus acquisition rate for vector	0.007 plants·day^-1^	[8]
*λ*	rate of loss of disease by vector	1 day^-1^	[7, 11]
*1/α*	mean dispersal distance	150 m	[36, 37]
*W*	vector population	200 individuals	[8, 33]
*ω*	vector natural death rate	0.12 day^-1^	[7, 11, 33]
*m*	vector migration rate	0.04 day^-1^	[33, 38]
*A*	attractive area of field	20 000 m^2^	-
-	number of fields	6000	http://kids.fao.org/agromaps/
-	chance of a grower trading for planting material	50%	[23, 36, 37, 39]
-	maximum trading partners	3 partners	[40]

aCertain parameters included for generality in the model are set to zero to simplify testing of hypotheses H1-H4. The effect of a non-zero value for *μ* is included in S2 Appendix, while *g* and *η* are set to zero as both could confound the effect of clean seed, and no reliable data exist for their values. Anecdotally, g is often presumed to be zero for subsistence growers.

We assume that the majority of growers are subsistence farmers, as is generally the case across the district. This implies that fields are harvested continuously at a constant rate over a period of months during the season, commencing after the first two months, with one replanting event at the start of each season (*μ* = 0, h>0). During this event, every field is replanted with planting material from one of three sources (from a grower’s own field, from material traded with neighbours, each informed by the level of infection in the previous season, or from uninfected material obtained from a clean-seed source). Additionally, roguing, while included for generality, is unlikely to be practiced by subsistence growers in the area and is therefore presumed to be absent. For alternative harvesting and replanting strategies, describing casual or commercial growing systems, we include additional results in S2 Appendix in the Supporting Information.

The size of a cassava field is taken to be 1.5 hectares (see, for example, [41]), and we presume an increase in affinity for whitefly of cassava fields over other areas. We presume that whitefly are a third again as likely to land on a cassava field as on a bare patch of land (see also S2 Appendix). We base the cassava field density of our model on the area of cassava harvested in Nakasongola district (10,000 hectares, http://kids.fao.org/agromaps/), and hence consider 6000 cassava fields (see S1 Appendix for details, where the modal distance of trading events is 500–1000 m). For the effect of changes in cassava field density see S2 Appendix. Whitefly migration is calculated from [38], using the total population to find the immigration rate at equilibrium, which we presume to be identical to emigration. To simulate the dispersal of the vector we use data from [37] and [42] for the dispersal of the sweet potato whitefly (on average, 50–700 m). We do not include the long-distance dispersal of whitefly that [42] observe in a second peak of migration, as this is not consistent with the exponential dispersal kernel that we have assumed. More importantly, however, whitefly may not remain infectious, or even survive, for the duration of these journeys [11, 43, 44]. However, we do show in S2 Appendix that the nature of our results is robust to the inclusion of this long-distance migration through a significant increase in the average distance travelled or the use of a fat-tailed dispersal kernel.

## Supporting information

S1 Appendix Host density calculations. (DOCX)

S2 Appendix Additional results. (DOCX)

S3 Appendix Trade dispersal. (DOCX)

S1 Video Dispersal of cassava brown streak viruses in Nakasongola district over 10 years, from 6 initially infected source fields. Dispersal of the pathogen between fields occurs through both the trade of infectious planting material and the between-field dispersal of infectious whitefly. Nakasongola district is shown in cream, where each circle represents a grower’s field, while triangles represent fields planted using certified clean planting material from a clean seed system. The legend describes the infection level in a field.
(AVI)

S2 Video Dispersal of cassava brown streak viruses in Nakasongola district over 10 years, from 6 initially infected source fields. Dispersal of the pathogen between fields occurs through trade only, with within-field dispersal of whitefly. Nakasongola district is shown in cream, where each circle represents a grower’s field, while triangles represent fields planted using certified clean planting material from a clean seed system. The legend describes the infection level in a field.
(AVI)

S3 Video Dispersal of cassava brown streak viruses in Nakasongola district over 10 years, from 6 initially infected source fields. Dispersal of the pathogen between fields occurs through between-field dispersal of whitefly only. Nakasongola district is shown in cream, where each circle represents a grower’s field, while triangles represent fields planted using certified clean planting material from a clean seed system. The legend describes the infection level in a field.
(AVI)

S4 Video Dispersal of cassava brown streak viruses in Nakasongola district over 10 years when infection pressure is high (70% of fields infected with 100% infection) and clean planting material is distributed to 10% of growers every season. Material is distributed in a fixed manner, to the same cluster of growers over successive seasons. Nakasongola district is shown in cream, where each circle represents a grower’s field, while triangles represent fields planted using certified clean planting material from a clean seed system. The legend describes the infection level in a field.
(AVI)

S5 Video Dispersal of cassava brown streak viruses in Nakasongola district over 10 years when infection pressure is high (70% of fields infected with 100% infection) and clean planting material is distributed to 10% of growers every season. Material is distributed in a fixed manner, to the same randomly dispersed growers over successive seasons. Nakasongola district is shown in cream, where each circle represents a grower’s field, while triangles represent fields planted using certified clean planting material from a clean seed system. The legend describes the infection level in a field.
(AVI)

S6 Video Dispersal of cassava brown streak viruses in Nakasongola district over 10 years when infection pressure is high (70% of fields infected with 100% infection) and clean planting material is distributed to 10% of growers every season. Material is distributed in a variable manner, to different randomly dispersed growers each season. Nakasongola district is shown in cream, where each circle represents a grower’s field, while triangles represent fields planted using certified clean planting material from a clean seed system. The legend describes the infection level in a field.
(AVI)

S7 Video Dispersal of cassava brown streak viruses in Nakasongola district over 10 years when infection pressure is high (70% of fields infected with 100% infection) and clean planting material is distributed to 10% of growers every season. Material is distributed in a variable manner, to different clusters of growers each season. Nakasongola district is shown in cream, where each circle represents a grower’s field, while triangles represent fields planted using certified clean planting material from a clean seed system. The legend describes the infection level in a field.
(AVI)

S8 Video Dispersal of cassava brown streak viruses in Nakasongola district over 10 years when infection pressure is high (70% of fields infected with 100% infection) and clean planting material is distributed to 10% of growers every season. Material is distributed in a variable manner, to different growers each season, expanding outwards from an initial cluster. Nakasongola district is shown in cream, where each circle represents a grower’s field, while triangles represent fields planted using certified clean planting material from a clean seed system. The legend describes the infection level in a field.
(AVI)
