# Supplementary material for: Spatial dynamics and control of a crop pathogen with mixed-mode transmission
Source: PLoS Comput Biol. 2017 Jul 26;13(7):e1005654. doi: 10.1371/journal.pcbi.1005654 (PMC5528833; doi:10.1371/journal.pcbi.1005654)
Supplement: S1 Appendix — (DOCX) [file pcbi.1005654.s001.docx]

S1 Appendix. Host density calculations

**

Figure S1.1: Location of Nakasongolo district in Uganda.

A population density model is used to distribute cassava production data per administrative unit (sourced from the LandScanTM 2011 Dataset, developed as part of the Oak Ridge National Laboratory Global Population Project at an approximately 1 km, 30''x30'', resolution. See http://www.ornl.gov/sci/landscan/). The model uses a country's sub-national census data and combines it with additional datasets including land cover, roads, urban and rural locations to redistribute the population according to a weighting scheme. It is assumed that the population living in urban extents is unlikely to be growing cassava, and these areas are masked out from our cassava density model. Urban extents for Africa are obtained from the Global Rural-Urban Mapping Project (v1) provided by the NASA Socioeconomic Data and Applications Center (SEDAC), hosted by the Center for International Earth Science Information Network (http://sedac.ciesin.columbia.edu/data/collection/grump-v1).

Information about cassava production serves as a proxy for distribution estimates. Annual cassava production in metric tonnes (mt) is derived from AgroMaps (http://kids.fao.org/agromaps/) for 2009, the most recent year available for Uganda. The first step of the calculations involves eliminating urban areas from the LandScan population raster data based on the GRUMP urban extent layer in ArcMap. The non-urban population number per administrative unit is then calculated. Further, the AgroMaps cassava production per administrative unit is divided by the total non-urban population per administrative unit to obtain production per capita. Finally, the LandScan population number in each pixel is multiplied by the production per capita in a given administrative unit to obtain cassava production per square kilometer. Information for Nakasongola province (see Figure S1.1) from the host distribution map in Uganda is then extracted for this study.

The host distribution map is used to estimate the likelihood of a given number of fields being located in each pixel. Once each field has been stochastically allocated to a particular pixel its location within that pixel is determined randomly (see Figure S1.2).


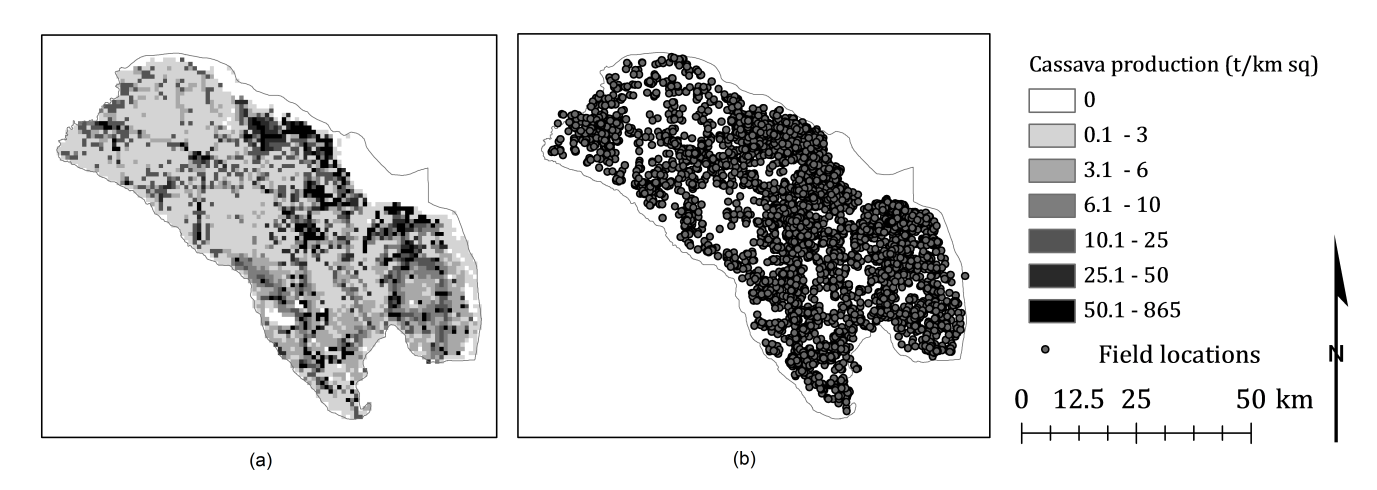


Figure S1.2: The (a) density of cassava and (b) simulated location of cassava grower's fields across Nakasongola district, central Uganda.

*Interpolation of results*

To present our model outcomes on a map of the district, we interpolate results using the ordinary kriging tool in ArcMap 10. Ordinary kriging is a widely used technique for interpolation distinct from traditional mathematical methods, introducing a stochastic model of spatial variation. Kriging expresses the spatial variation of an unknown variable by variogram and minimizing the prediction errors [1].

*References*

1. Oliver MA, Webster R. Kriging: a method of interpolation for geographical information systems. International Journal of Geographical Information Systems. 1990;4:313-32.
