# Supplementary material for: Spatial dynamics and control of a crop pathogen with mixed-mode transmission
Source: PLoS Comput Biol. 2017 Jul 26;13(7):e1005654. doi: 10.1371/journal.pcbi.1005654 (PMC5528833; doi:10.1371/journal.pcbi.1005654)
Supplement: S2 Appendix — (DOCX) [file pcbi.1005654.s002.docx]

S2 Appendix. Additional results

*Example*

Figure S2.1 shows an example of the dispersal of infection from one initially infected source field over time, through the movement of both infected whitefly and infected planting material.

(a)

(b)

(c)

(d)


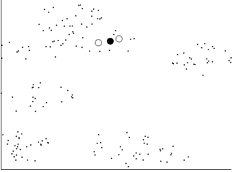

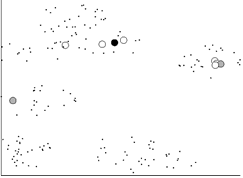

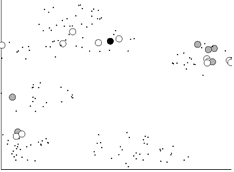

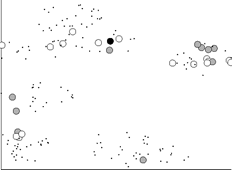


Figure S2.1: Example of the dispersal of CBSVs in a 7.5 x 10 km area of Nakasongola district over time from a single infected field (black circle). Dispersal of the pathogen to uninfected fields (black dots) occurs through either the exchange of planting material (grey circles) or whitefly between-field dispersal (white circles). Subplots show sequential time steps, after (a) 1, (b) 4, (c) 7 and (d) 10 years.

*Individual trading characteristics*

In Figure S2.2 we consider the impact of number of suppliers and loyalty to those suppliers at an individual (as opposed to population) level, comparing yields in different populations of growers for loyal and disloyal growers with many or few suppliers. We see that the incentive for an individual, aiming to minimise disease risk for her crops, is, as for the population as a whole, to be loyal and have few trading partners. In each case the infection level for loyal growers is lower than that for disloyal growers, and systems where growers have fewer suppliers contain lower levels of infection (the only exception is the average infection when the number of loyal growers in the district is high, in which case infection levels are so low that growers with more suppliers are in fact more likely to obtain uninfected material). However, this is not a guarantee of success, which depends on the sources that the grower is loyal to.

(b)

(a)


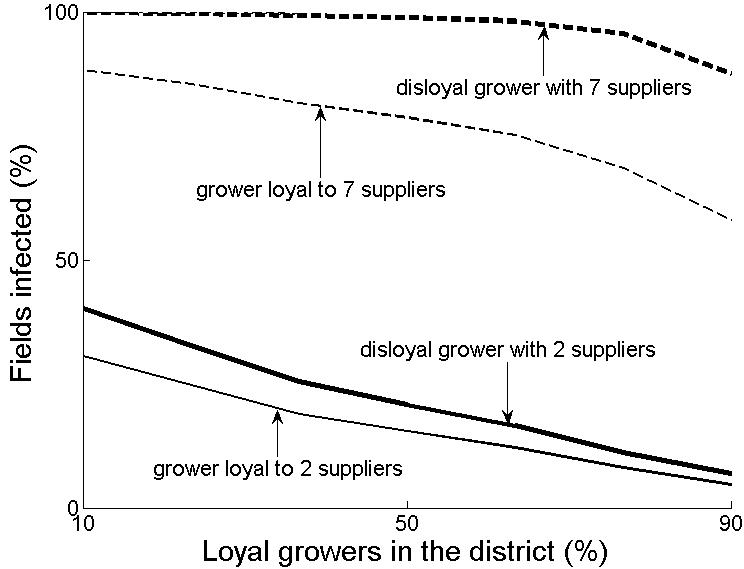

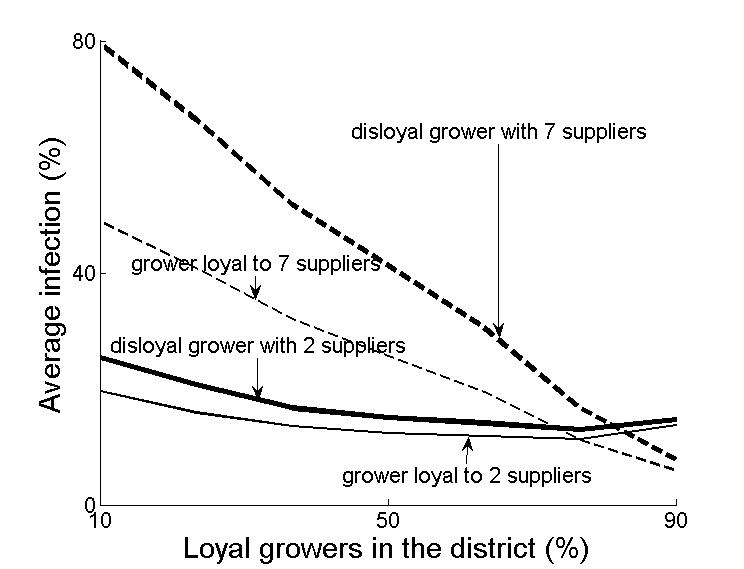


Figure S2.2: Mean (a) percentage of fields infected and (b) average infection in those fields in Nakasongola district after 30 years. Legends display the different scenarios considered, comparing the results, for a given percentage of loyal growers in the district, of loyal (thin lines) and disloyal (thick lines) growers in each scenario for a given maximum number of suppliers from whom growers may obtain cuttings, either 2 (solid lines) or 7 (dashed lines).

*Infection in clean material*

In Figure S2.3 we demonstrate the qualitatively consistent nature of our results on clean seed systems and trade restrictions when the clean material contains some level of infection. Here this is 10%, which is the maximum level of infection in seed sold to growers as recommended by guidelines proposed, and currently being appended to the Seed Act of Tanzania, by the 5CP project in conjunction with the Tanzania Official Seed Certification Institute (Dr. J.P. Legg, pers comm, 2^nd^ September 2015).

(b)

(a)


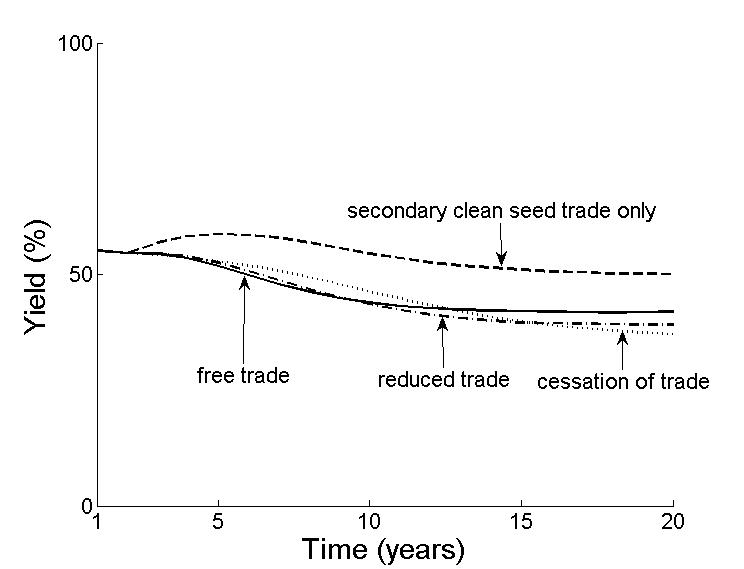

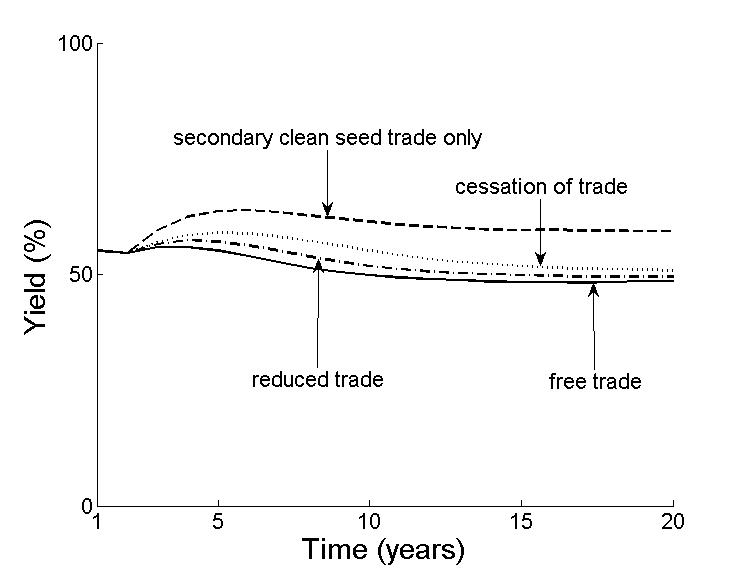


Figure S2.3: Average yield in Nakasongola district after 20 years, where 70% of fields are infected with 100% infection. “Clean” planting material from a clean seed system, containing 10% infection, is used by (a) 10% of the growers, distributed to the same growers over successive seasons or (b) 10% of the growers, distributed to different growers every season. Trade of planting material is allowed to continue as usual (solid lines), is reduced by 50% (dot-dashed lines), completely ceases, so that the pathogen can only be dispersed through the between-field dispersal of infectious whitefly (dotted lines) or is restricted so that only users of certified clean material in a particular season are licensed to distribute material at the end of that season (dashed lines). Each line is the mean of 100 realisations of the model. Results are analogous to Figure 4b, c in main text.

*Agronomic systems*

We illustrate the qualitatively consistent nature of our results for different agronomic systems in Figure S2.4. No matter how we vary significant parameters surrounding field management and the whitefly population, our results remain the same; trade is always more important for pathogen dispersal and it is always beneficial for growers across the district to remain loyal to only a few trading partners.

In addition, considering the source of infection for fields in Figure S2.4, 68% of infected fields are a result of trade in a, 73% in d, 74% in g, 66% in j, 64% in m and 99% in p. However, as previously a significant number of cases considering trade only do not result in an epidemic occurring; 0% of cases for a, 30% for d, 10% for g, 30% for j, 30% for m and 30% for p as well as 40% of cases when considering both trade and between-field whitefly dispersal.

Further to this, in Figure S2.5 we consider the qualitatively robust nature of our results on the introduction of a seed system for the same scenarios; the optimum yield is always obtained by varying the distribution of certified material to an expanding cluster of growers every year, then to different clusters of growers each year, followed by varying distribution to a dispersed group of growers, then distributing material to the same set of dispersed growers every year and finally distributing material to the same sets of clustered growers every year. In addition, a reduction in trade is only beneficial if clean planting material is distributed to different growers each season, while a maximum benefit is obtained if trade is restricted to the users of a clean seed system.

(o)

(n)

(m)

(j)

(k)

(l)

(g)

(h)

(i)

(f)

(d)

(e)

(c)

(b)

(a)


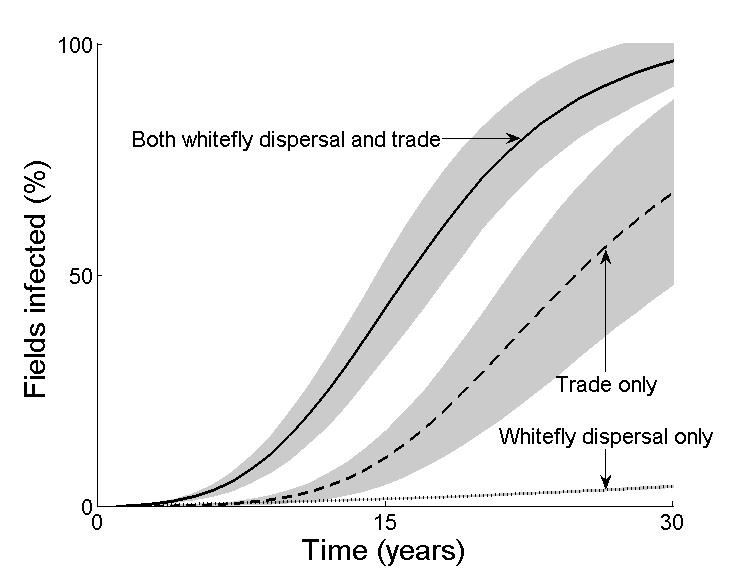

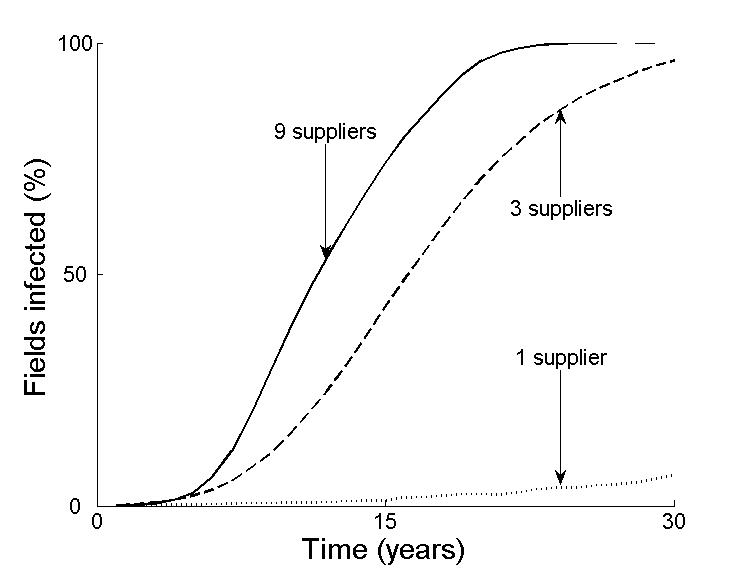

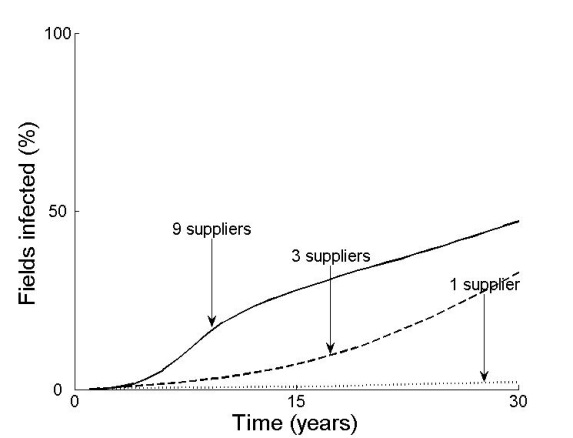

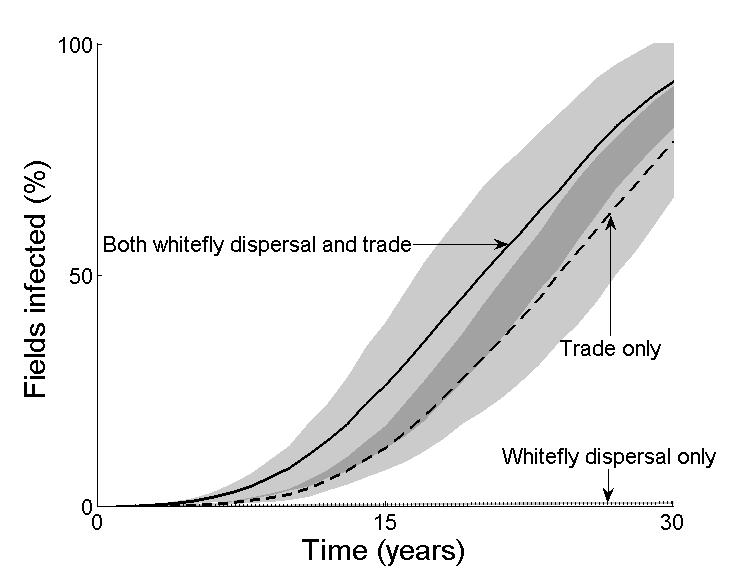

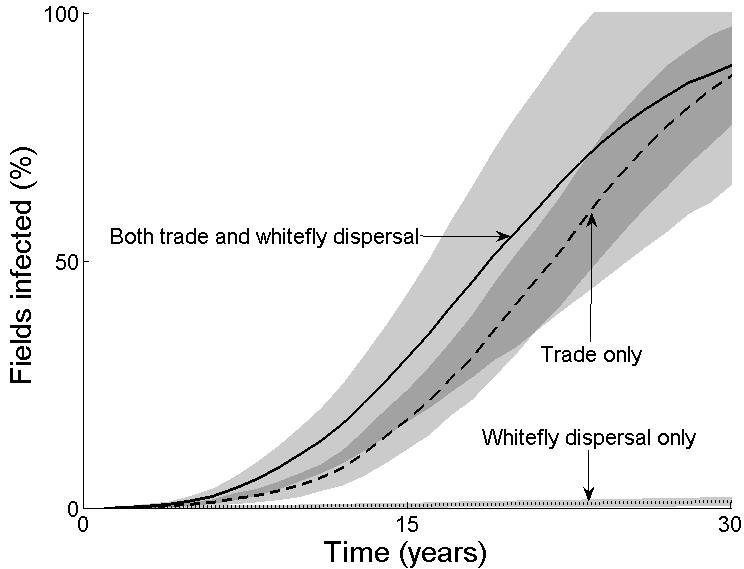

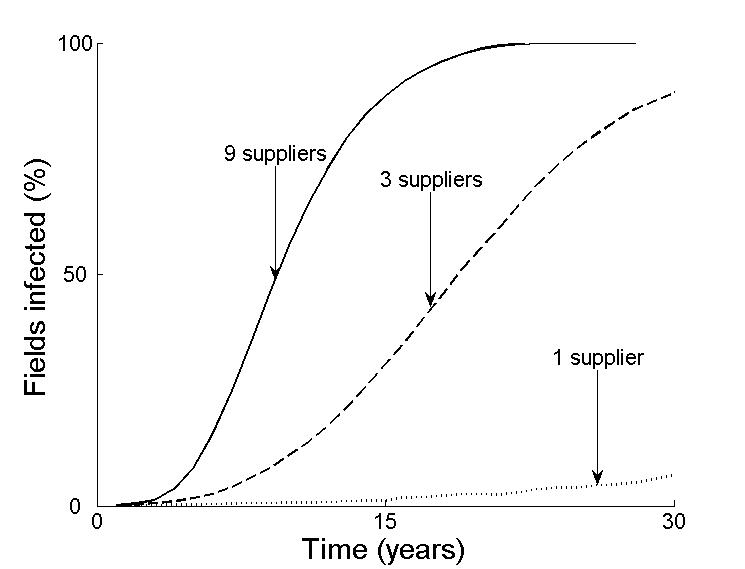

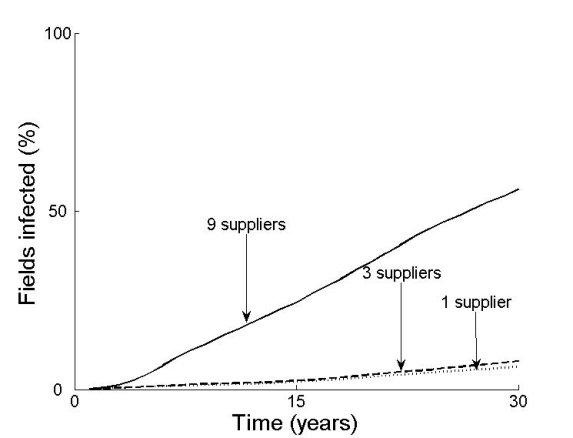

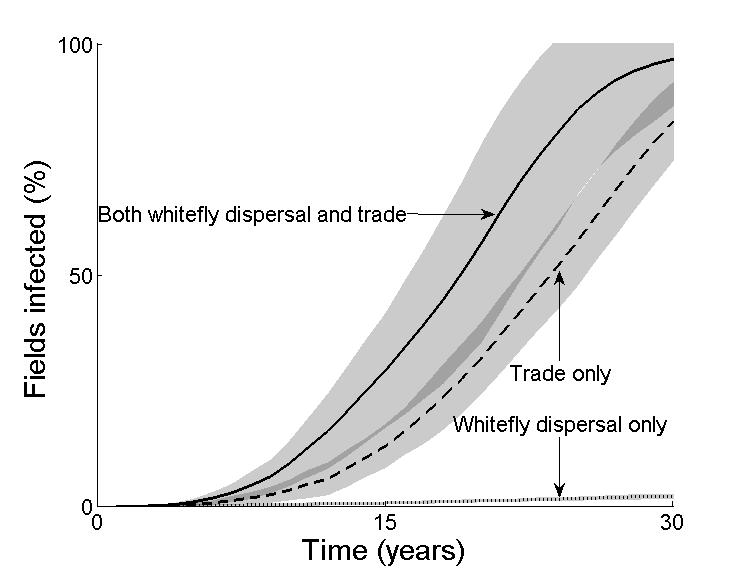

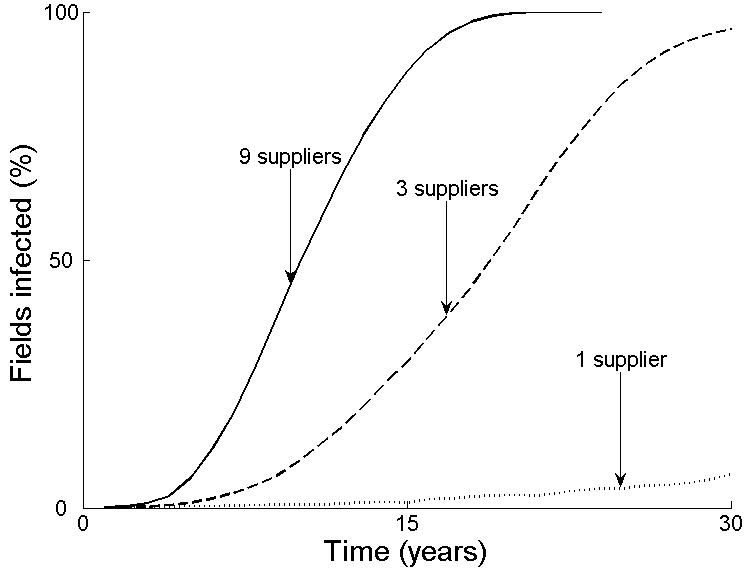

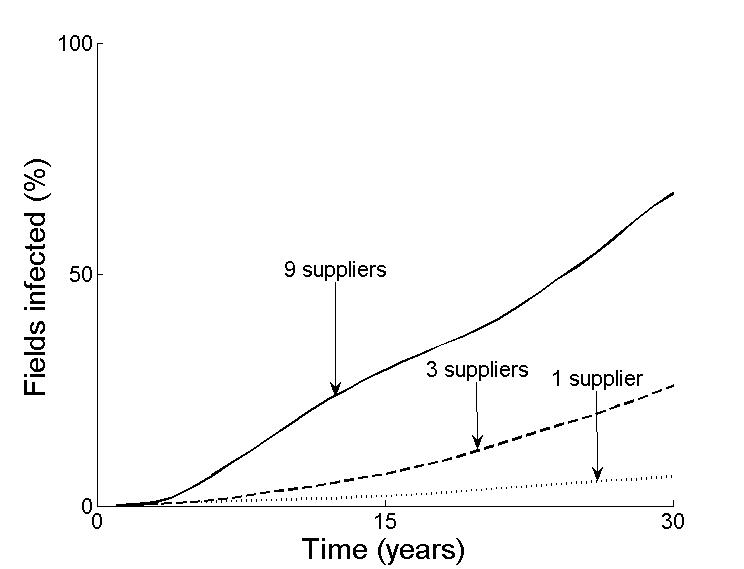

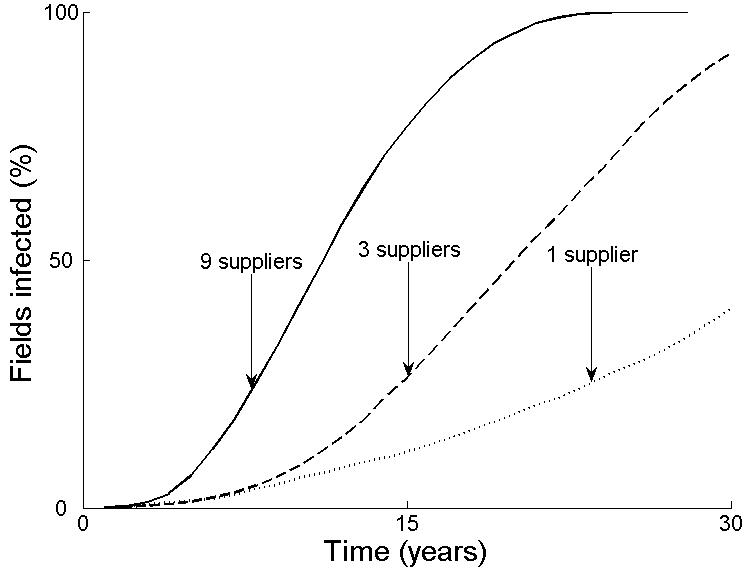

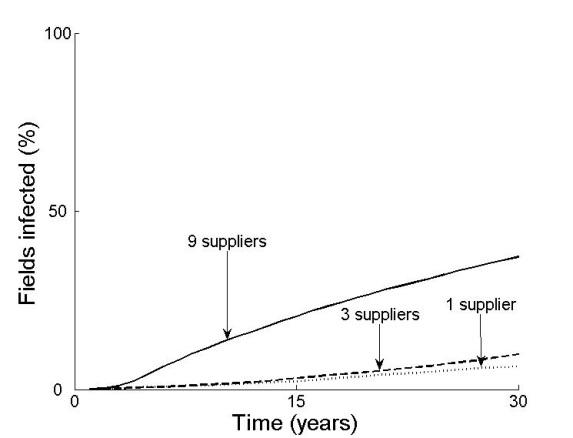

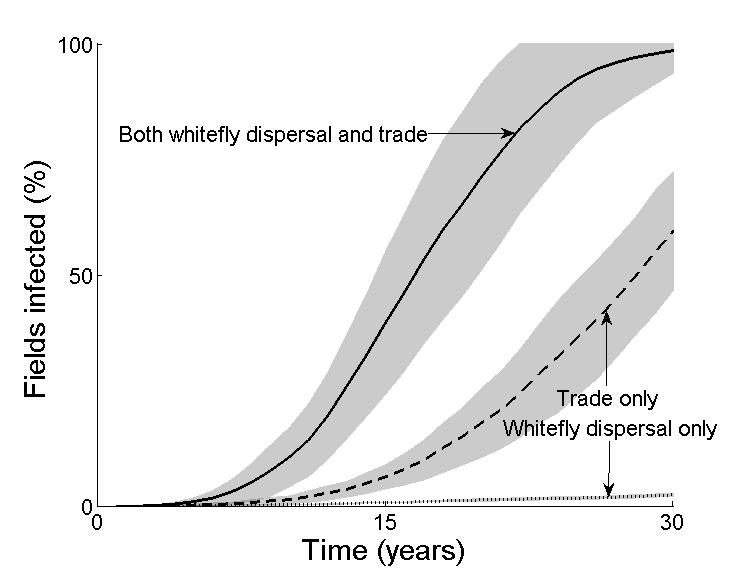

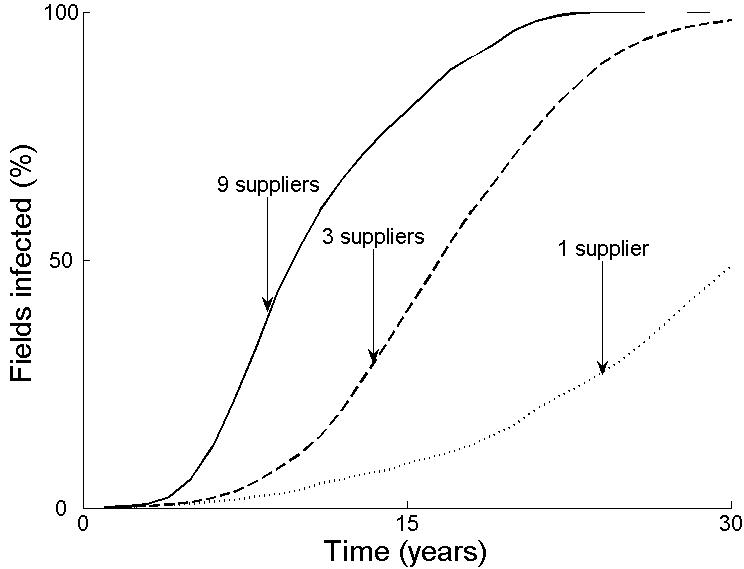

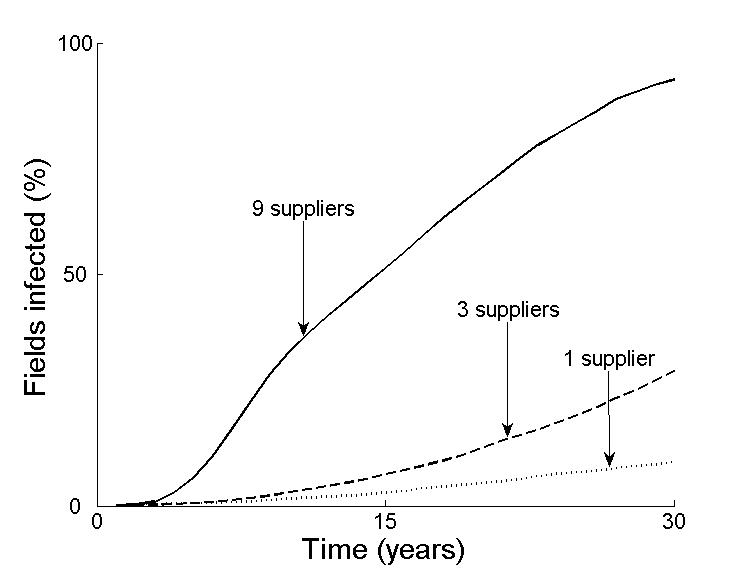


(p)

(r)

(q)


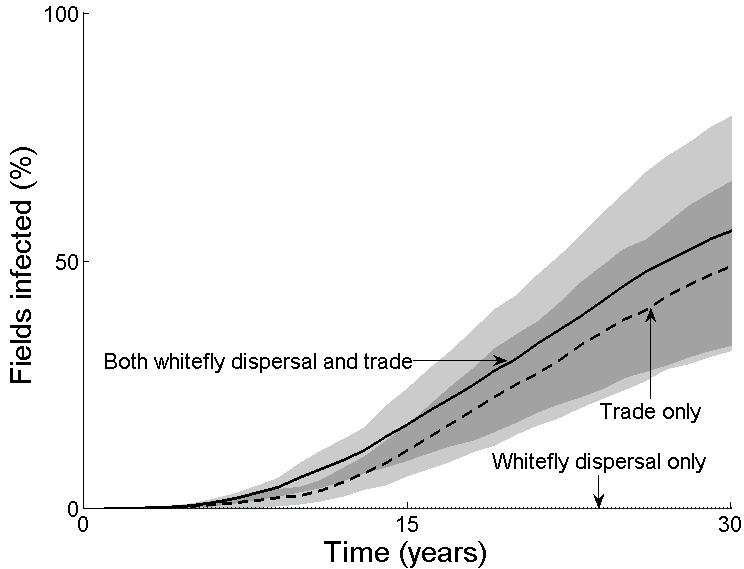

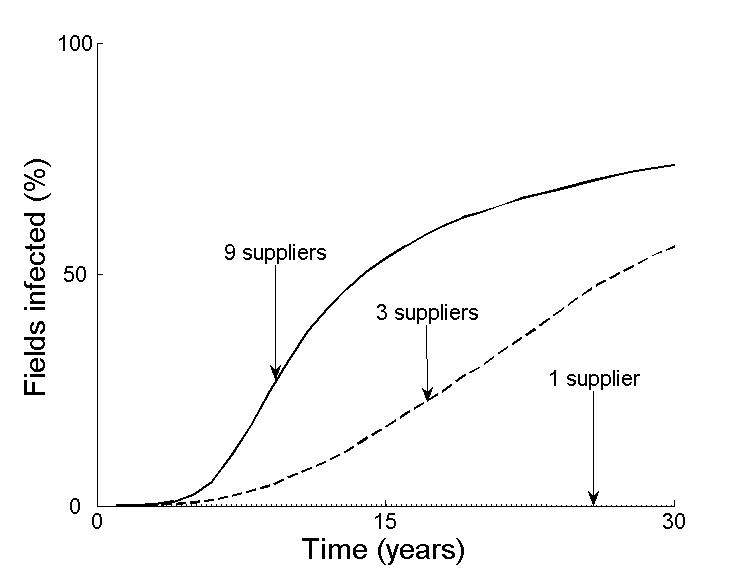

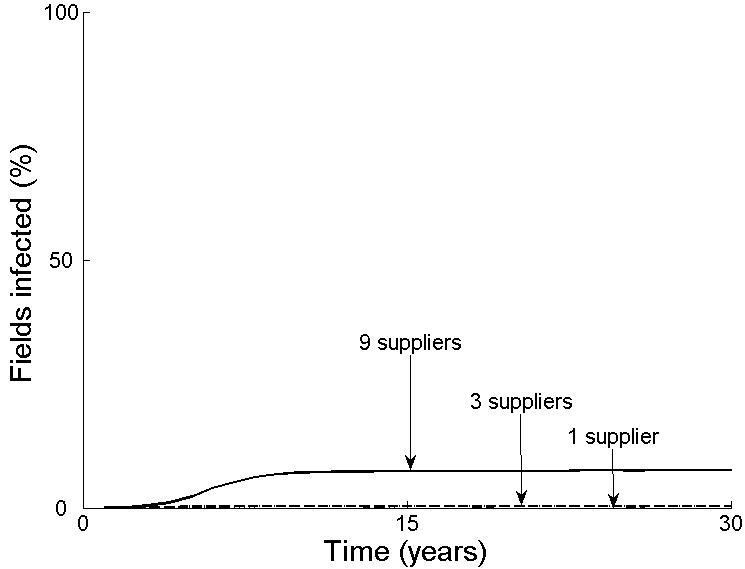


**Figure S2.4: Percentage of fields in Nakasongola district that are infected after 30 years. Each plot shows a different agronomic system to the main text. In a-c the distribution of fields across the district is randomised, and the field density is reduced to 1000 fields. In d-f the area of a field is increased to 10 hectares. In g-i the crop is continuously harvested and replanted throughout the season. In j-l the crop is harvested and replanted annually, at the end of each season. In m-o the whitefly population is increased to 500 whitefly per plant. In p-r the whitefly population is decreased to 5 whitefly per plant. Subplots a, d, g, j, m and p are comparable to Figure 2 in the main text, subplots b, e, h, k, and q to Figure 3b and subplots c, f, i, l, o and r to Figure 3a.**


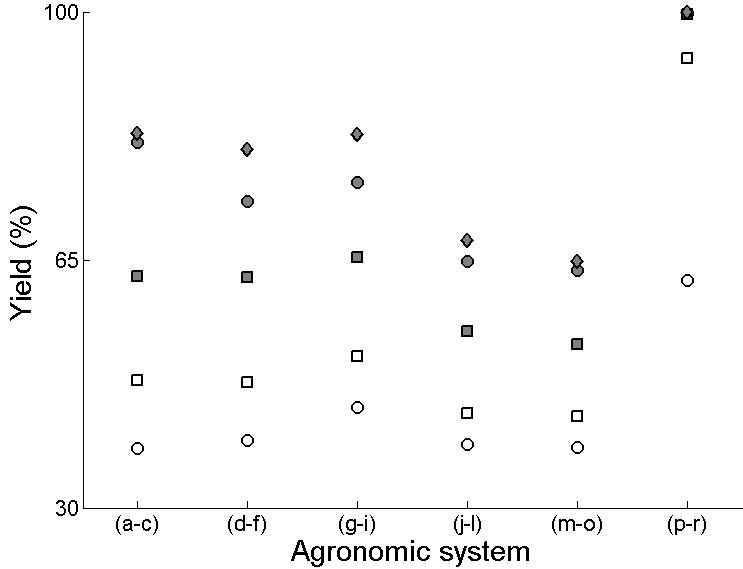

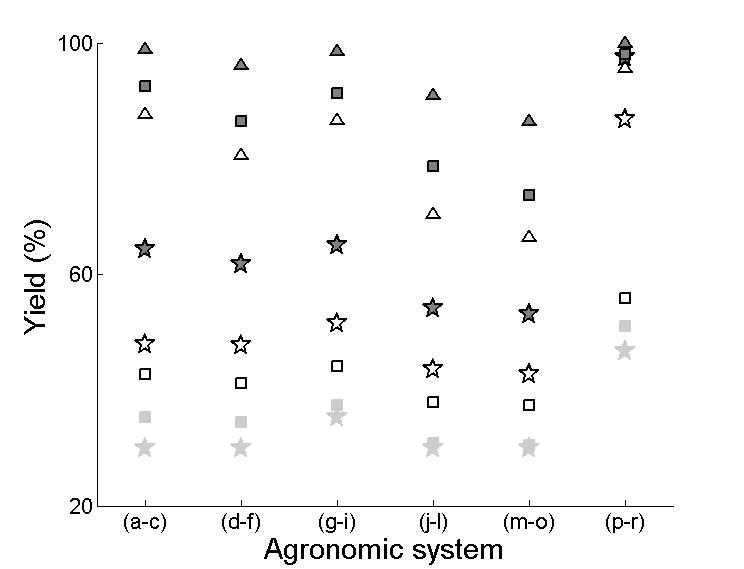


(a)

(b)

Figure S2.5: Average yield in Nakasongola district after 20 years, where 70% of fields are initially infected with 100% infection. Plots are comparable to (a) Figure 4 and (b) Figure 5 in the main text. In (a) clean planting material from a clean seed system is used by none of the growers (filled grey symbols), 10% of the growers, distributed to the same growers over successive seasons (open symbols), or 10% of the growers, distributed to different growers every season (filled dark symbols). Trade of planting material is allowed to continue as usual (stars), completely ceases (squares) or is restricted so that only users of certified clean material in a particular season are licensed to distribute material at the end of that season (triangles). In (b) clean material from a clean seed system is used by 10% of the growers, distributed to the same growers every season (open symbols), or by 10% of the growers, distributed to different growers every season (filled dark symbols). Material is distributed either to a cluster of growers (circles), at random (squares) or to a different group of growers each season expanding outwards from an initial cluster (diamonds). The agronomic systems are those described above in Figure S2.4.

*Improved material*

In Figure S2.6 we show that our results are also qualitatively consistent if the clean material distributed is tolerant or partially or completely resistant.


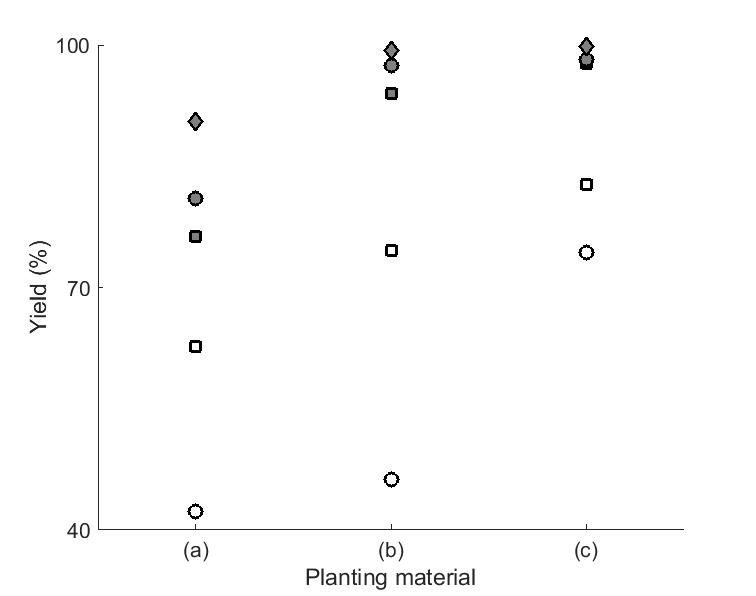

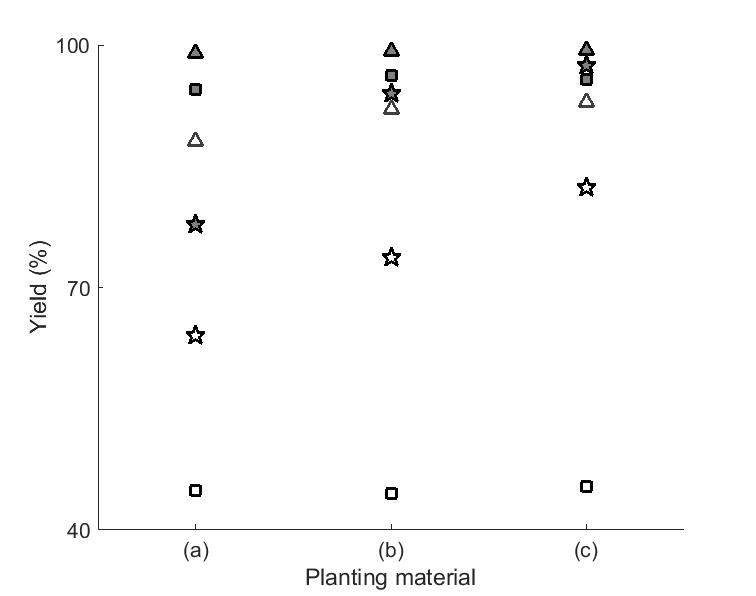


(a)

(b)

**Figure S2.6: Average yield in Nakasongola district after 20 years, where 70% of fields are initially infected with 100% infection. Subplots and symbols are comparable to Figure S2.5 above. Planting material type is (a) tolerant to disease, where the fraction of infectious plants that contributes towards yield is 60% as opposed to 30%, (b) partially resistant to disease, where the infection rate is reduced to** $\boldsymbol{\beta}_{\boldsymbol{p}}\boldsymbol{=}$**0.0007 as opposed to** $\boldsymbol{\beta}_{\boldsymbol{p}}\boldsymbol{=}$**0.007, or (c) completely resistant to disease, where** $\boldsymbol{\beta}_{\boldsymbol{p}}\boldsymbol{=}$**0.**

*Whitefly dispersal*

In Figure S2.7 and Figure S2.8 we show results for cases where whitefly between-field movement is altered; the mean dispersal distance is increased, the dispersal kernel is altered, the migration rate is increased or the rate of loss of infection is decreased. In all cases, our results remain qualitatively similar to previous. The exception to this is that for an altered dispersal kernel or migration rate, whitefly between-field dispersal and trade together result in lower levels of infection occurring than trade alone; this is due to the large standard deviation in the results, and the fact that the whitefly migrate out of a field too quickly and are unable to effectively increase infection within a field before dispersing.


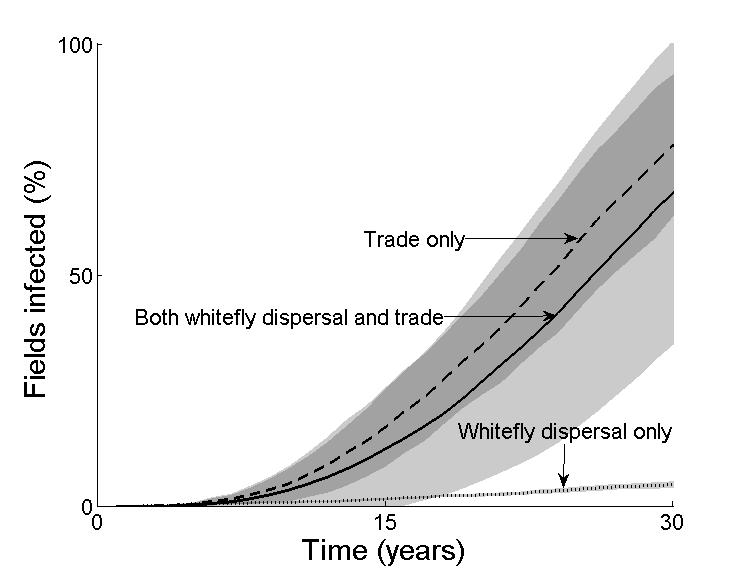

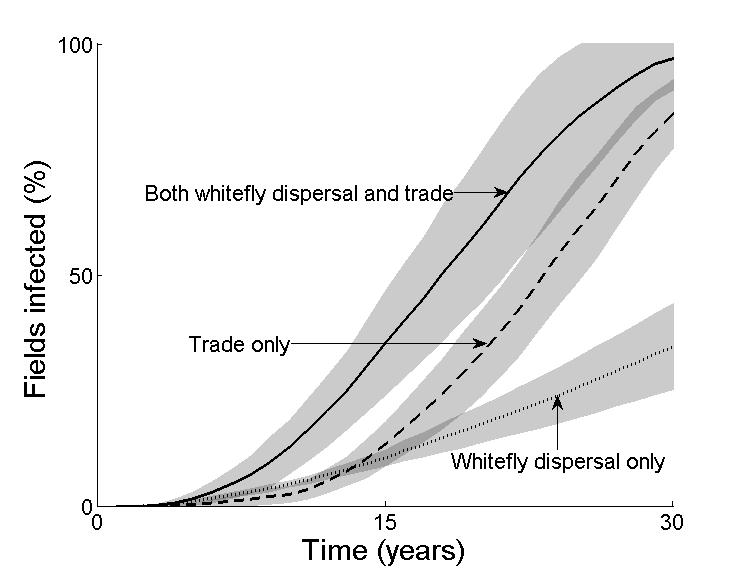


(a)

(b)


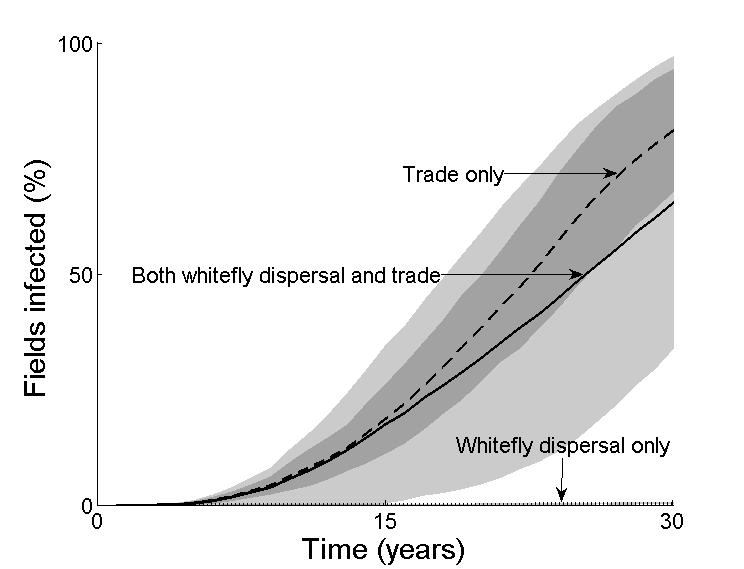


(c)


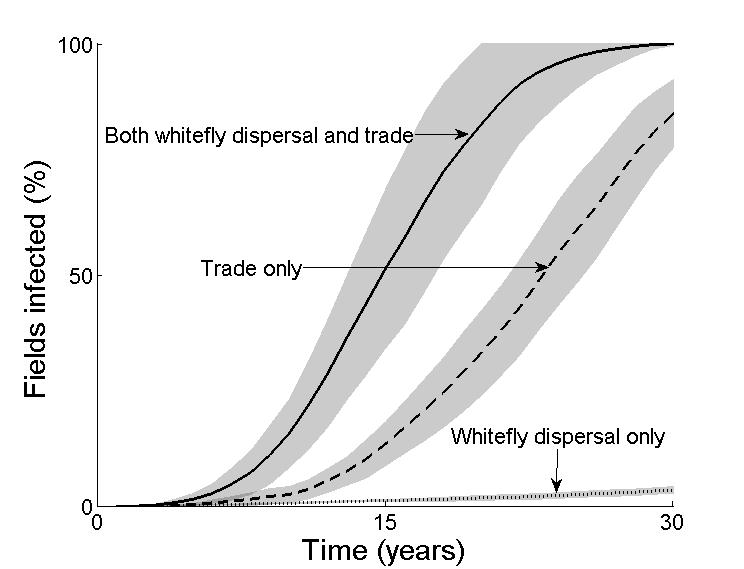


(d)

Figure S2.7: Percentage of fields in Nakasongola district that are infected after 30 years. Each plot shows a case where one parameter for whitefly movement has been altered. In (a) we increase the mean dispersal distance to 1.5 km. In (b) we fit a fat-tailed root dispersal kernel to the whitefly dispersal data ($\boldsymbol{\delta}_{\boldsymbol{ij}}\boldsymbol{= A B}\boldsymbol{e}^{\boldsymbol{-\psi\surd}\boldsymbol{d}_{\boldsymbol{ij}}}$ where $\boldsymbol{B=}\frac{\boldsymbol{\psi}^{\boldsymbol{4}}}{\boldsymbol{24}\boldsymbol{\pi}}$ and $\boldsymbol{\psi=\surd20}\boldsymbol{\alpha}$. See, for example, Madden, Hughes (1) and Demon, Cunniffe (2)). In (c) we increase the migration rate of the whitefly to 0.5 per day. Finally, in (d) we increase the length of time that the whitefly remains infectious for to 1 week. Results are analogous to Figure S2.4.


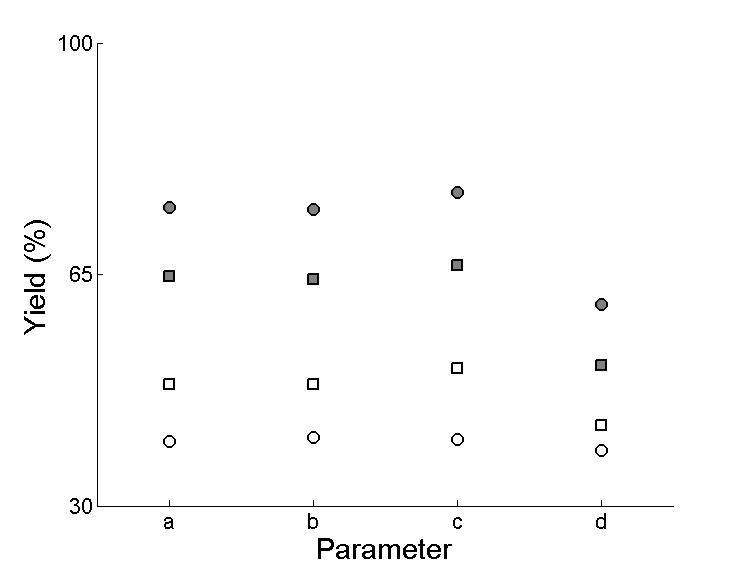


**Figure S2.8: Average yield in Nakasongola district after 20 years, where 70% of fields are infected with 100% infection. The plot is comparable to Figure S2.3b above, where each parameter is as described in Figure S2.7.**

*References*

1. Madden LV, Hughes G, Bosch Fvd. The study of plant disease epidemics. Madden LV, Hughes G, Bosch Fvd, editors2007. xiv + 421 p.

2. Demon I, Cunniffe NJ, Marchant BP, Gilligan CA, van den Bosch F. Spatial Sampling to Detect an Invasive Pathogen Outside of an Eradication Zone. Phytopathology. 2011;101(6):725-31. doi: 10.1094/phyto-05-09-0120. PubMed PMID: WOS:000290824400010.
