# Supplementary material for: Spatial dynamics and control of a crop pathogen with mixed-mode transmission
Source: PLoS Comput Biol. 2017 Jul 26;13(7):e1005654. doi: 10.1371/journal.pcbi.1005654 (PMC5528833; doi:10.1371/journal.pcbi.1005654)
Supplement: S3 Appendix — (DOCX) [file pcbi.1005654.s003.docx]

S3 Appendix. Trade dispersal

An exploratory data set from a recent small-scale pilot survey (23 respondents) in Eastern Zambia show that 43% of the time planting material was recycled from the previous crop in the same field. For trade transactions involving cutting movement, 52% of transactions occurred over distances <5 km, 27% over distances of 5-10 km, and 21% over distances >10km. We use these data to define trade probabilities between fields, where fields within a given distance range of a source are uniformly likely to trade with that source, up to a given maximum number of trades. The maximum number of trading partners in the survey matched that of Rohrbach and Kiala (1), with 48% of growers that traded having only one trading partner, 43% having two trading partners and 9% having 3 trading partners.

Additionally, we simulate trade relationships using a fat-tailed root dispersal kernel to rank neighbouring fields. This is given by $\rho_{ij}= r B e^{-\psi\surd d_{ij}}$ where $r\in[0,1\mathbf{]}, B=\frac{\psi^{4}}{24\pi}$ and $\psi=\frac{\sqrt{20}}{16800}$ for mean dispersal distance 16.8km taken from survey data [2, 3].

Results in both cases were qualitatively similar to the case of an exponentially decreasing likelihood of trade described in the main text (see Figures S4.1-3). We note in addition that traditional network metrics are unaffected by the relationships assumed here; for example, outdegree is predetermined by the maximum number of trade partners presumed, while connectedness and degree distribution are determined by this as well as the number of growers trading.

(e)

(f)

(d)

(a)

(b)

(c)


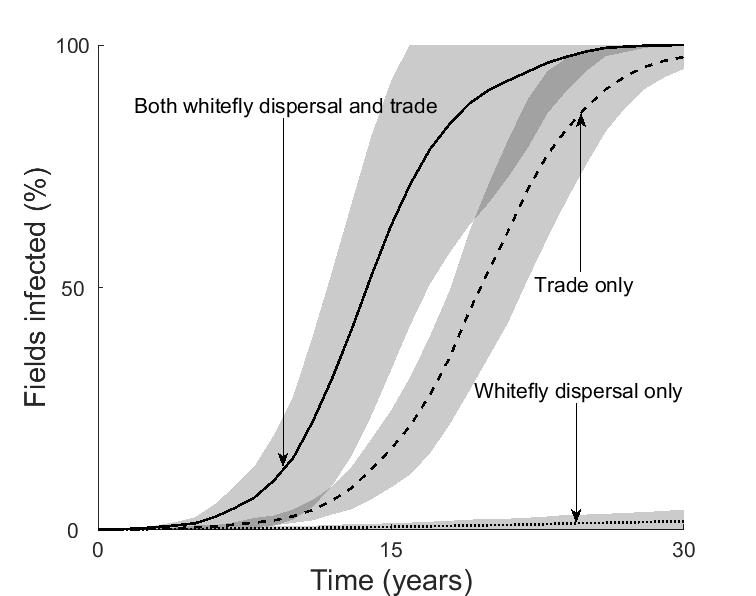

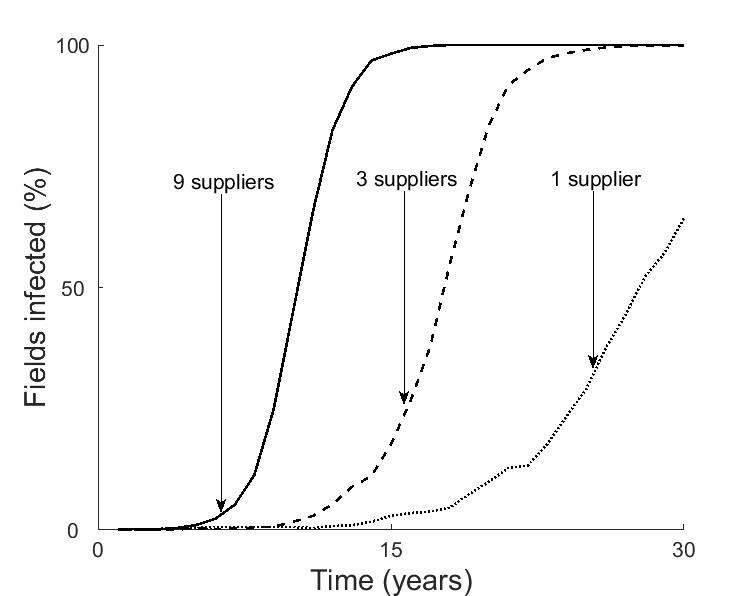

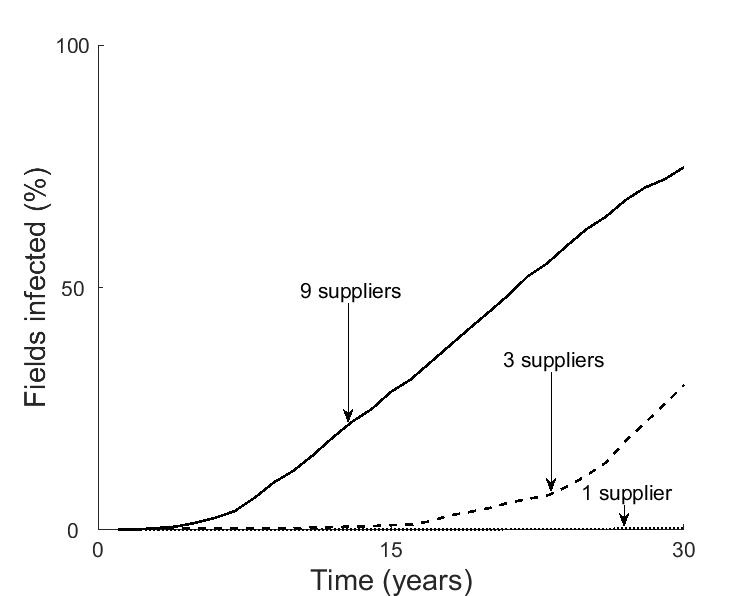

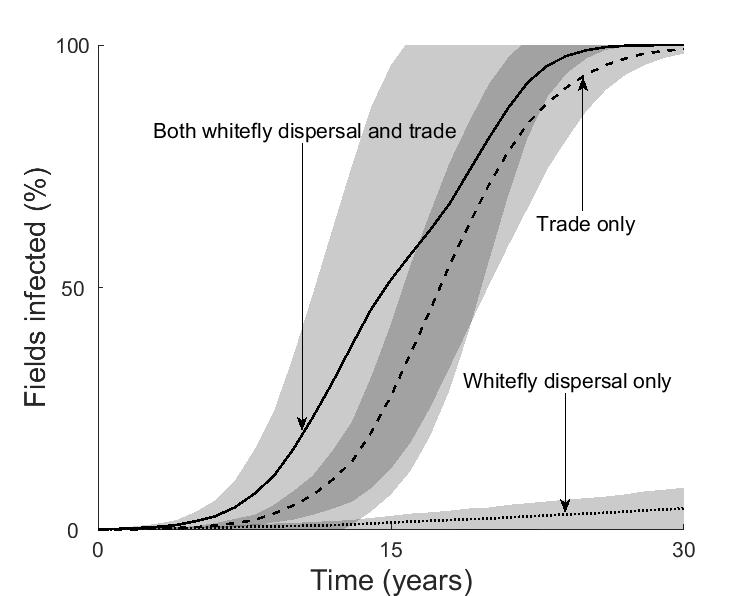

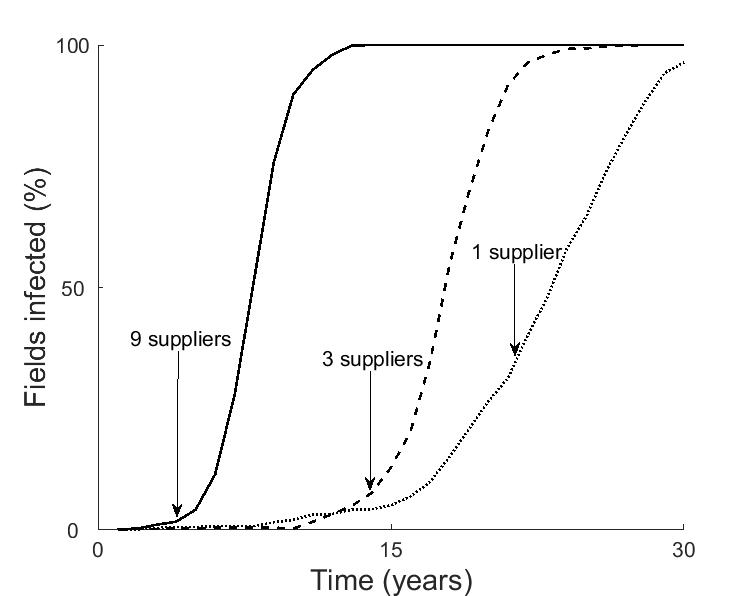

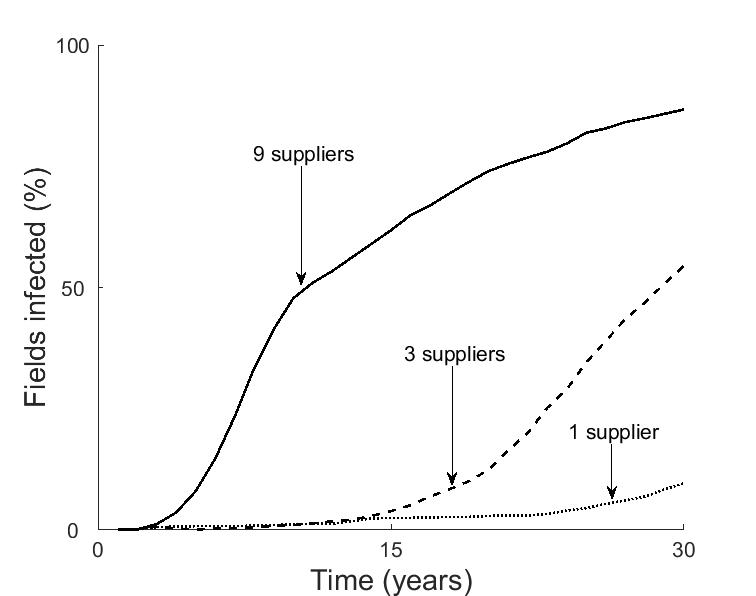


Figure S4.1: Percentage of fields in Nakasongola district that are infected after 30 years. In a-c trade takes place over a network simulated from the data described above. In d-f trade takes place over a network simulated using a thick-tailed probability distribution. Subplots a, d are comparable to Figure 2 in the main text, subplots b, e to Figure 3b and subplots c, f to Figure 3a. See also Figure S2.4.


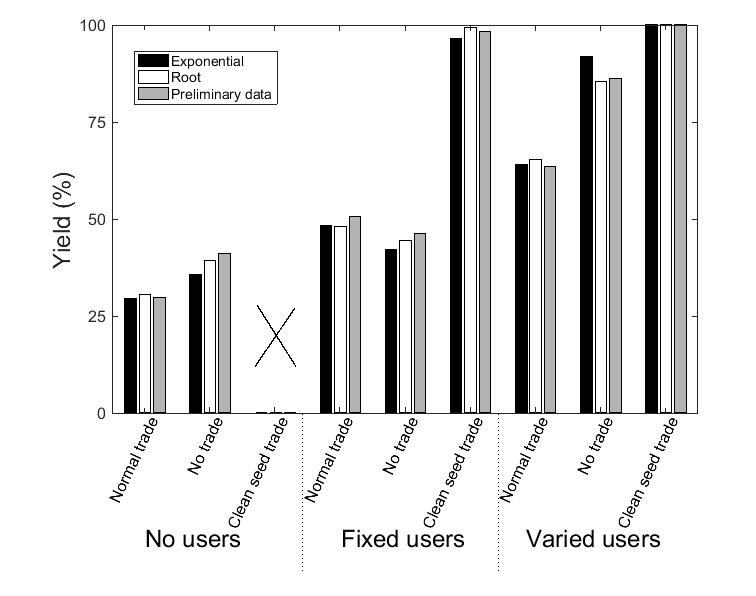


Figure S4.2: Average yield in Nakasongola district after 20 years, where 70% of fields are initially infected with 100% infection. We compare the effect of dispersal kernel used to describe stochastic trade interactions, based on an exponential (black) or root (white) dispersal kernel, or on an exploratory data set (grey). Clean planting material from a clean seed system is used by: none of the growers (No users); 10% of the growers, distributed to the same growers over successive seasons (Fixed users); or 10% of the growers, distributed to different growers every season (Varied users). Trade of planting material is allowed to: continue as usual (Normal trade); completely cease (No trade); or is restricted so that only users of certified clean material in a particular season are licensed to distribute material at the end of that season (Licensed trade).


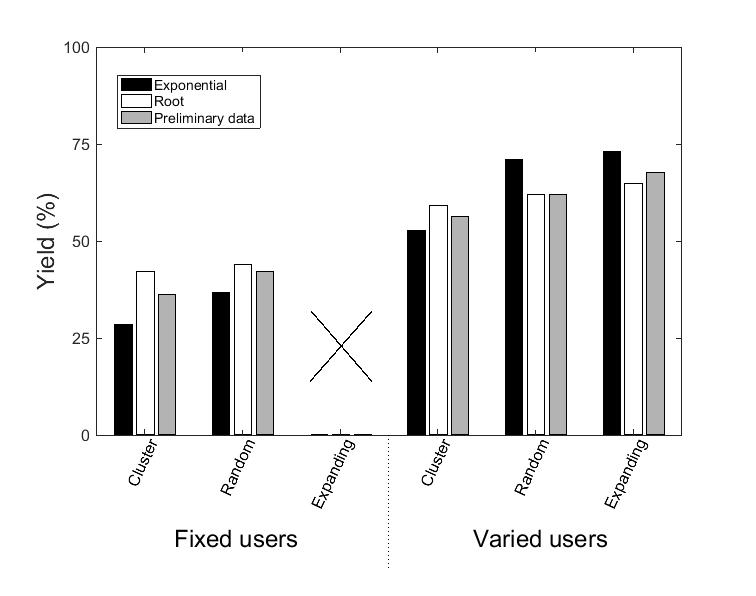


Figure S4.3: Average yield in Nakasongola district after 20 years, where 70% of fields are initially infected with 100% infection. We compare the effect of dispersal kernel used to describe stochastic trade interactions, based on an exponential (black) or root (white) dispersal kernel, or on an exploratory data set (grey). Clean material from a clean seed system is used by: 10% of the growers, distributed to the same growers every season (Fixed users); or by 10% of the growers, distributed to different growers every season (Varied users). Material is distributed either to a cluster of growers (Cluster), at random (Random) or to a different group of growers each season expanding outwards from an initial cluster (Expanding).

1. Rohrbach D, Kiala D. Development options for local seed systems in Mozambique. Working Paper Series no 5: Socioeconomics and Policy Program, International Crops Research Institute for the Semi-Arid Tropics. 2000:1-32.

2. Demon I, Cunniffe NJ, Marchant BP, Gilligan CA, van den Bosch F. Spatial Sampling to Detect an Invasive Pathogen Outside of an Eradication Zone. Phytopathology. 2011;101(6):725-31. doi: 10.1094/phyto-05-09-0120. PubMed PMID: WOS:000290824400010.

3. Madden LV, Hughes G, Bosch Fvd. The study of plant disease epidemics. Madden LV, Hughes G, van den Bosch F, editors2007. 421 p.
